# Using Student-Reported Digital Daily Logs and Human-Verified AI-Assisted Reflection Coding to Describe Clinical Experiences in Community-Based Clerkships: Exploratory Observational Mixed Methods Study

**DOI:** 10.2196/102390

**Published:** 2026-09-29

**Authors:** Naoto Ozaki, Kiyoshi Shikino, Nobuyuki Araki, Hiroshi Tajima, Hajime Kasai, Ikuo Shimizu, Kazuyo Yamauchi, Shoichi Ito

**Affiliations:** 1 Department of Community-Oriented Medical Education Graduate School of Medicine Chiba University Chiba Japan; 2 Health Professional Development Center Chiba University Hospital Chiba Japan; 3 Department of Medical Education Graduate School of Medicine Chiba University Chiba Japan; 4 Department of Medical Education School of Medicine Dokkyo Medical University Mibu, Tochigi Japan

**Keywords:** medical education, clinical clerkship, faculty development, community-based medical education, rural health services, medical students, clinical procedures, clinical skills, digital educational data, artificial intelligence, AI

## Abstract

**Background:**

In Japan, the 2021 revision to the Medical Practitioners Act formally supported students’ participation in supervised clinical practice. However, monitoring day-to-day clinical experiences across community-based clerkship sites remains challenging. Student-reported digital daily logs and reflection sheets provide complementary descriptive information across such sites.

**Objective:**

This study aimed to investigate whether a digital educational data workflow, combining student-reported daily clinical activity logs with human-verified artificial intelligence (AI)–assisted reflection coding, can be used to describe clinical learning experiences across distributed community-based clerkship sites.

**Methods:**

In this exploratory observational study, we explored variations across placement contexts, including whether sites included community-based attending physicians (CAPs) trained in the university faculty development (FD) program. Data from 112 fifth-year medical students who had completed 3-week community-based clinical clerkships at sites with trained CAPs (n=34) or at sites without trained CAPs (n=78) were analyzed. Students recorded daily experiences of 50 Model Core Curriculum (MCC)–based clinical activities using a Google Spreadsheet–based digital daily log. The primary outcome was the number of student-reported examination-based, lower-risk clinical activity types recorded as performed at least once. Log completion, defined as the presence of any substantive daily log entry, was assessed across eligible student-days, and a classification-independent sensitivity analysis examined all 50 activity items. Reflection sheets were deductively coded according to MCC learning objectives using a human-verified AI-assisted preliminary sorting and candidate-coding workflow. Furthermore, exploratory sensitivity analyses examined the role of the placement context.

**Results:**

Daily log entries were available for 1491 (99.6%) of 1497 eligible student-days. Students at sites with trained CAPs had more student-reported examination-based, lower-risk clinical activity types recorded as performed at least once than those at sites without trained CAPs. No statistically significant difference was observed for basic clinical and emergency procedures. The classification-independent analysis across all 50 items showed a similar pattern. Reflection sheet analysis yielded 4097 textual units. Placement category was closely associated with medically underserved placement, home visit exposure, and baseline rural self-efficacy.

**Conclusions:**

The digital workflow yielded nearly complete student-reported daily data and described variation in clinical experiences across clerkship sites. These differences should be interpreted as variation across placement contexts rather than evidence of an independent effect of supervision by trained CAPs. Reflection sheets provided complementary descriptive information, and the AI component required human verification.

## Introduction

Performing clinical procedures and examinations during clinical clerkships is an important part of preparing medical students for clinical work [1]. Students need opportunities to observe physicians and perform clinical tasks under supervision. However, previous studies have highlighted gaps between the procedural experiences students want, the experiences they receive, and their confidence or competence at graduation [2,3]. These gaps may be larger in rural and community-based settings, where educational resources and faculty time are limited [4].

Medical education in Japan has moved toward greater student participation in clinical care [5]. A 2021 revision to the Medical Practitioners Act formally allowed medical students to perform procedure- and examination-related practices under supervision [5]. This change was intended to support participation-based clinical clerkships. However, in practice, students still spend substantial time observing rather than performing clinical tasks. Recent national surveys in Japan suggest that active student engagement remains limited [6].

Community hospitals and clinics play an important role in clinical education. However, they often work with limited staff, busy clinical schedules, and less formal educational infrastructure than university hospitals [7,8]. Although these settings offer authentic patient encounters and close contact with community care, their educational value depends on how supervisors invite students into clinical work. Evidence from longitudinal integrated clerkships suggests that continued supervision, patient care, and curriculum can support learner- and patient-centered education through coaching, progressive entrustment, and repeated feedback [9-13]. Therefore, community-based placements require supervisors who create graded opportunities for students to participate safely in care.

Faculty development (FD) for community-based attending physicians (CAPs) is one possible approach. CAPs often decide whether a student only observes a consultation or takes the history, performs a physical examination, assists with an investigation, or participates in treatment. FD programs for community-based clinical teachers can support teaching skills, assessment, feedback, interprofessional collaboration, and local teaching culture [14,15]. Prior FD initiatives included interactive workshops, longitudinal programs, feedback and reflection, workplace-based projects, communities of practice, and institutional support [14,15]. In this study, the presence of CAPs trained in the university FD program (hereafter, “trained CAPs”) was treated as a contextual characteristic among several placement factors rather than an independently testable intervention.

Distributed community-based clerkships provide diverse clinical learning opportunities. However, monitoring students’ participation across multiple sites remains challenging. Conventional end-of-rotation evaluations may not capture item- or week-level variations in students’ clinical experiences. However, digital daily logs can provide a structured method for capturing student-reported learning opportunities in real time, particularly when recorded items are aligned with a national competency or curriculum framework [16]. Their interpretation depends on implementation quality, including completion, delayed entries, reminder procedures, and external verification.

Large-scale student reflection data can complement structured clinical activity logs; however, they are resource intensive to be coded consistently [17,18]. Recent studies suggest that large language models can support qualitative coding, although their performance varies according to prompt design, coding approach and granularity, and contextual complexity, making human verification important [17-20]. Existing medical education studies have compared ChatGPT-driven and human-led qualitative analyses and applied ChatGPT to medical students’ reflective responses [18,20]. However, to the best of our knowledge, detailed reports of human-verified artificial intelligence (AI)–assisted preliminary sorting and candidate-coding workflows that deductively map medical students’ reflections to hierarchical national competency frameworks and quantify AI-human concordance across code levels remain limited. Therefore, transparent reporting of such workflows is important [21].

This study used Google Spreadsheet–based daily logs to collect Model Core Curriculum (MCC)–based student-reported procedure and examination experiences during a 3-week community-based clinical clerkship. We further used a human-verified AI-assisted preliminary sorting and candidate-coding workflow to organize deidentified reflection sheets according to the MCC-based educational framework. Recent literature has highlighted the potential of generative artificial intelligence (Gen AI) tools in medical education and the need for expert verification and transparent reporting [21-23]. Therefore, we conducted a formative, exploratory evaluation of a digital educational data workflow combining student-reported daily logs and human-verified AI-assisted deductive coding of reflection sheets. We described the implementation quality and informational contribution of the workflow and explored variation in student-reported clinical experiences across placement contexts, including the presence of trained CAPs. The reflection sheet component provided complementary descriptive context rather than explaining the mechanisms underlying the differences in activity counts.

## Methods

### Study Design

We adopted a pragmatist paradigm, focusing on practical questions relevant to community-based clinical education and the monitoring of student-reported learning experiences across clerkship sites [24,25]. We conducted an exploratory observational mixed methods study incorporating quantitative digital log analysis and deductive content analysis of students’ reflection sheets. The quantitative component was the primary analysis and described student-reported clinical experiences, while exploring variation across placement contexts, including the presence of trained CAPs. The reflection sheet analysis provided a complementary descriptive context by mapping students’ learning accounts with predefined MCC-based codes [26]; it was not designed to explain mechanisms or validate the digital log counts. Integration occurred through a joint display that aligned major quantitative findings with MCC-coded reflection patterns and the resulting integrated descriptive interpretation [27]. Reporting and methodological rigor were informed by the Good Reporting of A Mixed Methods Study (GRAMMS) checklist (Multimedia Appendix 1) [28].

### Setting and Participants

All eligible fifth-year medical students at our institution who participated in a community-based clinical clerkship from September to November 2024 were included. Before this 3-week clerkship, students completed 1 year of clinical clerkship at the university hospital. The clerkship cultivated a comprehensive attitude toward patients and community residents and helped students analyze the current state of community medical care, identify issues in local health care, and consider ways to develop and improve community care scientifically and practically.

Community-based clerkships were conducted at 38 institutions across 9 medical districts, including institutions in areas with physician shortages. Allocation was determined by student preference and a needs assessment, considering educational impact and clerkship readiness [29,30]. Since these factors may influence students’ opportunities to participate in clinical procedures and examinations, placement should be interpreted as nonrandom rather than as allocation to an intervention. Of the 112 students, 34 (30.4%) were placed at sites with trained CAPs and 78 (69.6%) at sites without such trained CAPs.

### FD Program and CAPs

CAPs were considered trained in the university FD program if they had been enrolled in the mandatory program for at least 12 months before the community-based clerkship and were actively involved in supervising students during the clerkship. A site was categorized as including trained CAPs if at least one such CAP actively supervised students during the clerkship; otherwise, it was categorized as a site without trained CAPs. Notably, the latter category does not indicate the absence of FD, supervisor training, teaching workshops, or educational experience among local supervisors.

The FD program was offered by the affiliated university. It was designed to build a shared educational culture within community sites, support community-oriented medical education, and enhance regional health care capacity. In community-based clinical education, CAPs are expected to provide direct student instruction and mentorship and develop and maintain local educational infrastructure [31,32]. The FD program consisted of 2-hour weekly sessions delivered primarily online, with in-person sessions held approximately once per month. Across the 38 available session agendas reviewed for this study, the program incorporated approximately 76 scheduled contact hours. Participation was mandatory for enrolled CAPs. The overall attendance rate was 99.3% on a person-session basis, indicating high and relatively consistent exposure to the FD curriculum among participating CAPs. Sessions were facilitated mainly by faculty members from the affiliated university responsible for community-based medical education and education of health professions, with topic-based contributions from CAPs and invited speakers. The program did not rely on a single standardized textbook or commercial teaching package; rather, sessions were delivered through lectures, workshops, practical exercises, peer sharing, and structured reflection.

Program content included curriculum design and MCC alignment, site-specific goal setting, workplace-based assessment, observation and feedback, microteaching, coaching, simulation-based education, and peer learning. Delivery fidelity was monitored through session agendas, attendance records, documentation of session outputs, and self-assessment and reflection forms. However, the extent to which each FD component was implemented in daily clinical supervision at each site was not directly observed or quantitatively assessed. Hence, this study did not attribute student-reported activity patterns to specific FD components. Additional details are provided in Multimedia Appendix 2.

### Quantitative Data Collection and Analysis

#### Daily Records of 50 Clinical Activities

Students documented their daily clinical experiences in a Google Spreadsheet. For each of the 50 MCC-based items, students selected one of three categories: performed, observed/simulated, or not performed. Before the clerkship, all students received standardized orientation regarding these classifications. The 50 clinical activities were selected based on the MCC for Medical Education in Japan and the Guidelines for Participatory Clinical Clerkship (Multimedia Appendix 3) [33,34]. Although the response categories were informed by the general concept of graduated participation and entrustment, they did not measure formal entrustment levels [35,36].

Faculty members regularly monitored the spreadsheet for missing, delayed, or inconsistent entries. The same university-level monitoring procedure was applied across all placement sites, and a standardized weekly reminder was sent to the students. Students were asked to complete or clarify entries, when necessary. Local supervisors did not independently verify each student-entered item; therefore, the data were analyzed as student-reported logs. The primary quantitative outcome was the number of student-reported examination-based, lower-risk clinical activity types recorded as performed at least once during the 3-week clerkship, using the author-defined category described later. This outcome captured only the diversity of student-reported activity types; it did not measure activity frequency, competence, entrustment, or supervisor-confirmed performance.

#### Implementation and Quality Control of the Digital Daily Log Workflow

For the retrospective assessment of implementation quality, an eligible student-day was defined as a day on which the student was scheduled to participate in the clerkship. Days of absence, holidays or site closure, and days without scheduled clerkship were excluded. Daily log completion was defined as the presence of any substantive daily log entry on an eligible student-day, whether in the reflection section, the clinical activity section, or both. Thus, the reported completion rate indicates that substantive content was entered for that day; it does not indicate completion of the clinical activity section or of all 50 clinical activity items. Item-level completeness was not used to define the reported completion rate.

The precise frequency and duration of delayed entries and the number or proportion of entries modified after faculty review were not stored as structured process indicators and could not be quantified reliably. No standardized item-level audit or partial supervisor confirmation procedure was implemented. Clinical Clerkship E-Portfolio of Clinical Training (CC-EPOC) had not yet been introduced for off-campus community-based clerkships at our institution in 2024. Therefore, CC-EPOC, the Mini-Clinical Evaluation Exercise (Mini-CEX), Entrustable Professional Activities (EPA)–related records, other workplace-based assessment data, and systematic supervisor-confirmed logs were unavailable for triangulation.

For analysis, we grouped these activities into two author-defined categories. First were examination-based, lower-risk clinical activities, which included medical interviews, selected physical examinations, medical record documentation, and position change/transfer. Some closely related physical examination items were integrated because they were commonly performed and reported as part of the same clinical assessment in community-based clerkships. Position change and transfer were included in this category because, although listed in the MCC, they were noninvasive patient care and functional assessment activities. Second were basic clinical and emergency procedures, which included activities derived from MCC and emergency-related procedures. This classification was exploratory and hypothesis generating. Furthermore, it was neither an official MCC classification nor an externally validated taxonomy.

### Participant Flow and Baseline Characteristics

All 112 students participating in the community-based clerkship were included in the quantitative phase. No formal a priori sample size calculation was performed, as this was an exploratory observational study using a fixed cohort comprising all eligible fifth-year medical students who participated in the clerkship during the study period. Therefore, the sample size was determined based on the number of eligible students and available clerkship placements, rather than a prespecified power calculation.

All students completed baseline surveys before the clerkship. Data on demographic characteristics, academic background, regional quota admission, and baseline rural self-efficacy were collected. Baseline rural self-efficacy was assessed using the rural self-efficacy scale developed by Kawamoto et al. [37] for Japanese medical students. The scale comprises 15 items rated on a 4-point Likert scale and includes four factors: work preferences, evaluation of rural practice, evaluation of rural living, and personal character. The theoretical total score ranges from 15 to 60, with higher scores indicating stronger rural self-efficacy and stronger orientation toward rural practice. Regional quota students were admitted through a prefecture-linked admissions pathway that provided a scholarship of JPY 10.8 million (~US $70,101) over the 6-year medical program and required 9 years of postgraduation service as physicians within the prefecture, including 2 years in medically underserved areas. Assignments to medically underserved areas were determined using the Physician Uneven Distribution Index [38,39].

### Qualitative Data Collection and Analysis

#### Daily Reflection Sheets

On scheduled clerkship days, medical students submitted an electronic reflection sheet addressing three prompts: what they had learned, what they could not do, and what they wished to learn next [40]. The number of eligible clerkship days varied among students because of holidays, site schedules, and absences; therefore, a fixed total of 15 days per student was not assumed.

#### Deductive Content Analysis

The qualitative component mainly examined how procedure- and examination-related learning objectives appeared in students’ daily reflections. The reflection sheets were divided into textual units and deductively assigned to MCC-based learning objectives [33,34]. The MCC is organized hierarchically into four tiers. The first tier comprises 10 broad competency domains, including Clinical Skills (CS), Problem-Solving (PS), and Interprofessional Collaboration (IP). The second tier divides these domains into 37 competency areas. The third tier comprises 125 specific learning categories, including medical interviews (CS-01-01), physical findings (CS-01-02), investigation techniques (CS-03-01), and treatment techniques (CS-03-02). The fourth tier comprises 595 detailed learning objectives nested within the third-tier categories [41].

In this study, final human-verified coding, frequency summaries, and weekly site category comparisons used third-tier codes. AI-human concordance was evaluated at the first-, second-, and third-tier levels. Although the initial custom GPT prompt allowed fourth-tier suggestions, these codes were not included in the final analytic dataset or analyses. They were treated as procedure- and examination-related categories. Since this coding was based on predefined MCC learning objectives, the reflection sheet analysis described students’ written learning content. One medical education faculty member (author NO) performed the primary coding, while another faculty member (author KS) reviewed the dataset as a supervisory second rater. Discrepancies were resolved through discussion.

#### Human-Verified AI-Assisted Preliminary Sorting and Candidate-Coding Workflow

Gen AI (ChatGPT and OpenAI) supported preliminary sorting and generation of candidate MCC codes for textual units based on students’ reflection sheets. The AI tool was used after the reflection sheets had been deidentified and divided into textual units. No student names, identification numbers, grades, or other personal identifiers were entered into the tool. No fine-tuning was performed. Since the tool was used through the ChatGPT custom GPT interface, model parameters, such as temperature and top-p, were not manually specified or recorded. AI-assisted workflow reporting was guided by the GAMER framework [21].

AI-generated candidate codes were used only as preliminary support. After the AI-assisted preliminary sorting and candidate-coding, two human researchers (NO and KS) reviewed the AI suggestions, checked the original deidentified textual units, and independently determined the final MCC codes. NO reviewed the AI-generated candidate codes against the original deidentified textual units and the MCC coding framework. KS supervised the process and reviewed the coded data as a second rater. Disagreements or uncertainties were resolved through discussion. Thus, the final analytic dataset was based on human-verified coding. This approach was informed by recent discussions on AI-assisted qualitative and content analysis [17,19]. Prompt templates, corrective prompts, representative outputs, and examples of AI-supported coding are provided in Multimedia Appendix 5. The workflow was not prospectively evaluated for efficiency, time savings, workload reduction, or usability.

A total of 4097 textual units were extracted from the reflection sheets. For the AI-human concordance analysis, duplicate coding records derived from the same textual unit were collapsed into a single analyzable unit. Units for which AI-generated candidate codes and human coding decisions could be directly compared were included in this analysis. Thus, 3752 unique analyzable units were used for the AI-human concordance analysis. At the third-tier MCC code level, complete agreement was 24.2% (909/3752), and at least one code overlapped at 37.4% (1404/3752). At the second-tier MCC code level, complete agreement was 44.6% (1675/3752), and at least one code overlapped at 61.8% (2319/3752). At the first-tier domain level, complete agreement was 58.7% (2202/3752), and at least one code overlapped at 76.8% (2880/3752). These initial concordance metrics characterized the performance of AI-generated candidate codes. Final coding reliability was assessed between the two human reviewers, and interrater agreement for the final MCC-based coding was excellent (κ=0.97).

### Joint Display

We constructed a joint display, aligning major quantitative findings with corresponding MCC-coded reflection patterns and the resulting integrated descriptive interpretation in order to describe how the digital log data and written reflection data converged or diverged when representing students’ clinical experiences. However, it did not explain causal mechanisms, differences between placement contexts, or validate the digital log counts.

### Statistical Analysis

Descriptive statistics summarized participant characteristics and outcomes. Continuous variables were reported as medians (IQRs), and categorical variables were reported as frequencies and percentages. Differences between the two site categories were assessed using Mann-Whitney *U* tests for continuous or ordinal variables and chi-square or Fisher’s exact tests for categorical variables. Effect sizes were calculated using rank-biserial correlation coefficients and interpreted according to standard thresholds [42]. Bias-corrected and accelerated 95% CIs for the rank-biserial correlations were estimated using 200,000 bootstrap resamples, with resampling performed separately within the two categories. Furthermore, as a classification-independent sensitivity analysis, we compared the total number of student-reported clinical activity types recorded as performed at least once across all 50 MCC-based items.

To examine whether weekly changes differed between the two site categories, we fitted a Poisson generalized estimating equation (GEE) model including site category, week, and the site category × week interaction, accounting for repeated measures among students. A Gaussian GEE model was fitted as a sensitivity analysis. Since the two site categories differed substantially in placement context, we developed a conceptual diagram to clarify hypothesized contextual overlap and reporting processes involving the presence of trained CAPs, medically underserved placement, home visit exposure, site type, site readiness/local educational culture, baseline rural self-efficacy, regional quota admission, sex, and student-reported clinical experiences (Multimedia Appendix 4).

Exploratory sensitivity analyses used Poisson regression models with robust SEs for the primary outcome, adjusting for home visit exposure, baseline rural self-efficacy, regional quota admission, sex, and site type. Since the presence of trained CAPs and medically underserved placement were strongly associated, with few students in the off-diagonal combinations, medically underserved placement was examined in separate contextual models. A model including both variables was fitted only to assess potential instability arising from sparse off-diagonal cells. Negative binomial models were further fitted because the outcome was count data, with the possibility of overdispersion. Mixed-effects models, facility-level cluster-robust SEs, and region-level clustering were not used as primary analyses because each facility included only 1-4 students and the resulting small clusters made these approaches unreliable for estimating an independent FD-related association. As previous-year or pre-FD student-level digital log data were unavailable for the first year after FD program implementation, before-and-after and difference-in-differences analyses were not performed. All analyses were conducted using R version 4.3.1 (R Foundation for Statistical Computing) and Python 3.12.

### Ethical Considerations

This study was approved by the Institutional Ethics Board of Chiba University (approval number: 3425). Participation was voluntary, and all students provided informed consent before data collection. All data collected were anonymized to protect privacy and confidentiality. Participants did not receive compensation for participation.

## Results

### Implementation and Quality of the Digital Daily Log Workflow

After excluding absences, holidays or site closure, and days without scheduled clerkship, 1497 eligible student-days were identified. Substantive daily log entries were available for 1491 (99.6%) student-days (99.6%); the remaining 6 (0.4%) student-days had no substantive entries. Delayed-entry duration and postreview correction counts could not be quantified from the retained data, and no standardized supervisor audit was conducted.

### Participant Overview and Group Allocation

A total of 112 fifth-year medical students were included in the analysis. Table 1 summarizes baseline student and placement characteristics for students placed at sites with or without trained CAPs. The two site categories differed in key placement context variables, including medically underserved placement, home visit exposure, and baseline rural self-efficacy. These differences were important contextual confounders when interpreting the findings.

**Table 1:** Characteristics of community-based clinical clerkship students (N=112).

Variables			Without FD^a^-trained CAPs^b^ (n=78)^c^	With FD-trained CAPs (n=34)		*P* value
Female, n (%)			25 (32.1)	9 (26.5)		.66
Regional quota student, n (%)			11 (14.1)	8 (23.5)		.28
Rural self-efficacy score at baseline, median (IQR)			41.50 (37.00-45.00)	46.00 (41.25-49.00)		.006
**Clerkship site, n (%)**						
	Clinic	25 (32.1)		12 (35.3)	.36	
	Hospital	42 (53.8)		22 (64.7)	.07	
	Home visit service	11 (14.1)		0	.006	
	Medically underserved area	4 (5.1)		33 (97.1)	<.001	
Exposure to home visit service, n (%)			43 (55.1)	29 (85.3)		.002
**Assigned clerkship site characteristics, n (%)**						
	Hosts early-year medical students	46 (59.0)		31 (91.2)	<.001	
	Hosts postgraduate clinical junior residents	56 (71.8)		30 (88.2)	.09	
	Has certified clinical supervisors of junior residents	64 (82.1)		34 (100)	.005	

^a^FD: faculty development.

^b^CAP: community-based attending physician.

^c^Sites without trained CAPs do not indicate the absence of other educational training.

### Classification of MCC-Based Clinical Activities

The 50 MCC-based clinical activities were classified into 23 (46%) examination-based, lower-risk clinical activities and 27 (54%) basic clinical and emergency procedures (Multimedia Appendix 3). Figures 1 and 2 compare the median number of student-reported clinical activity types recorded as performed at least once during the 3-week clerkship period.

**Figure 1:**
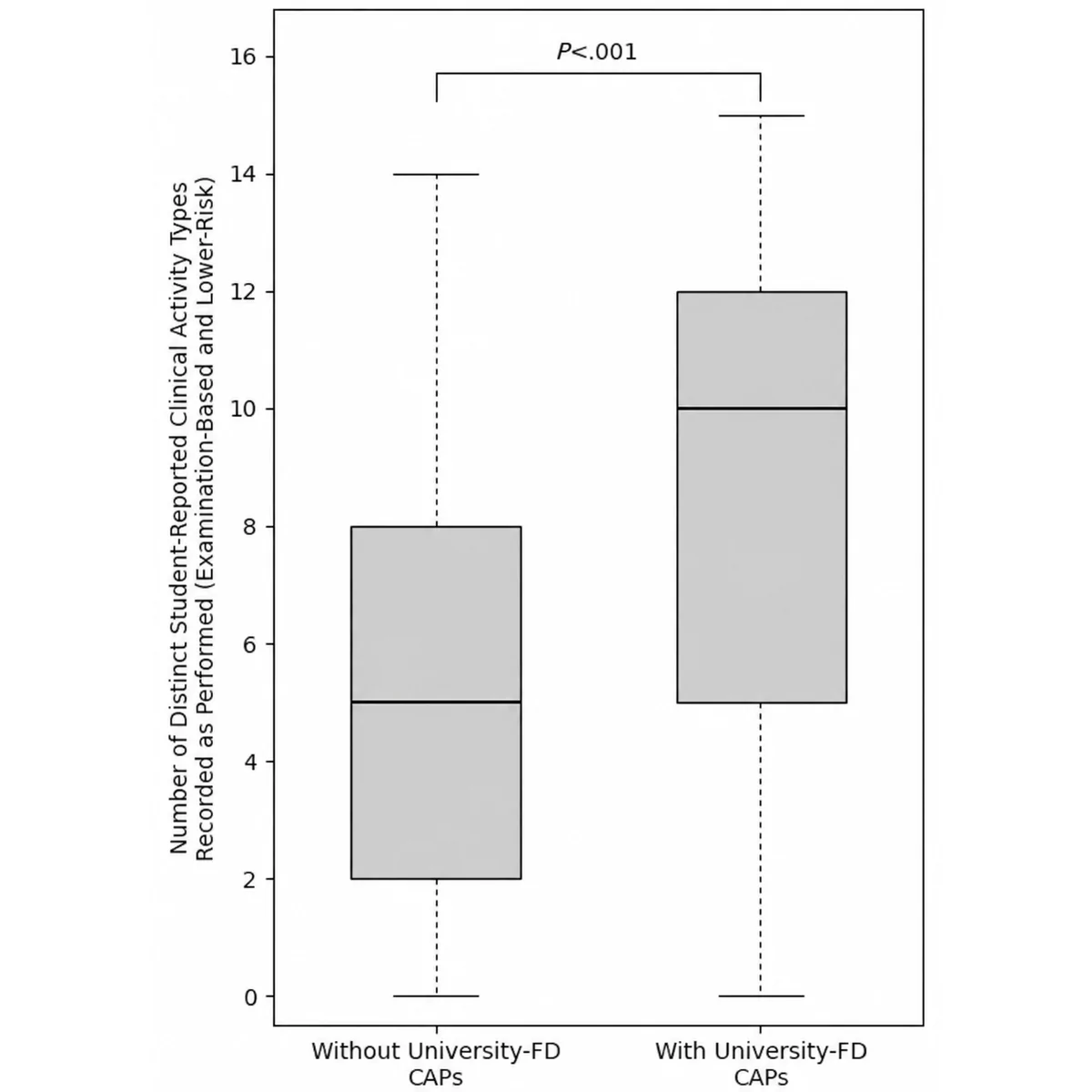
Number of student-reported examination-based, lower-risk clinical activity types recorded as performed at least once during the 3-week community-based clinical clerkship (23 items); horizontal lines indicate medians (*P*<.001). Students placed at sites with trained CAPs were compared with those at sites without CAPs. CAP: community-based attending physician; FD: faculty development.

**Figure 2:**
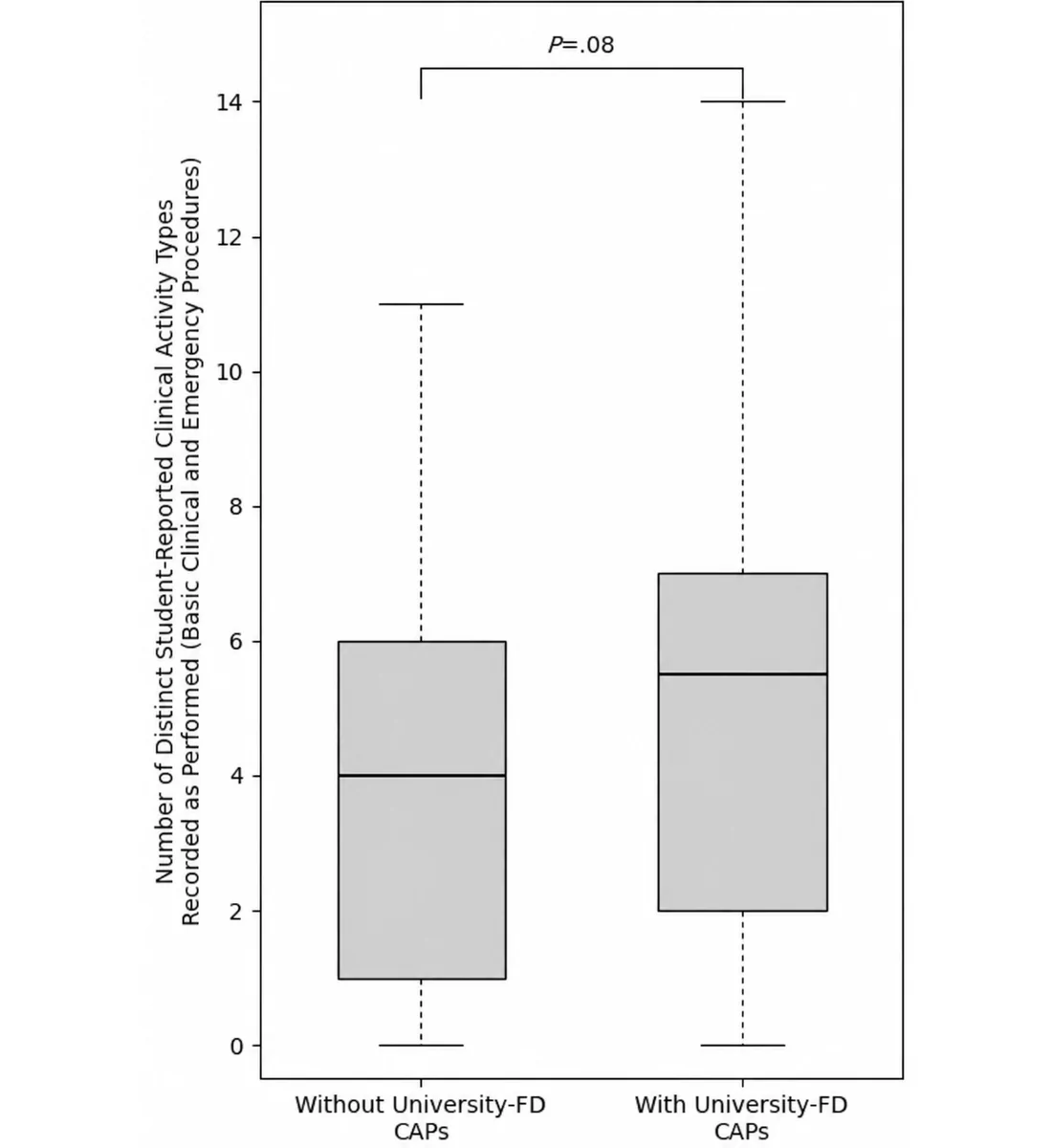
Number of student-reported basic clinical and emergency procedure types recorded as performed at least once during the 3-week community-based clerkship (27 items); horizontal lines indicate medians (*P*=.08). Students placed at sites with trained CAPs were compared with those at and sites without trained CAPs. CAP: community-based attending physician; FD: faculty development.

#### Comparison of Examination-Based, Lower-Risk Clinical Activities

Students placed at sites with trained CAPs had a higher number of student-reported examination-based, lower-risk clinical activity types recorded as performed at least once than those placed at sites without trained CAPs (median 10, IQR 5-12, vs median 5, IQR 2-8; Mann-Whitney *U*=1924.0; *P*<.001; rank-biserial correlation, r=0.45, 95% CI 0.21-0.64).

#### Comparison of Basic Clinical and Emergency Procedures

Students at sites with trained CAPs had a higher number of student-reported basic clinical and emergency procedure types recorded as performed at least once than those at sites without trained CAPs (median 5.5, IQR 2-7, vs median 4, IQR 1-6; Mann-Whitney *U*=1601.5; *P*=.08; r=0.21, 95% CI –0.04 to 0.43). The difference between site categories was not statistically significant.

### Classification-Independent Analysis Across All 50 Items

In the classification-independent sensitivity analysis, students at sites with trained CAPs had a higher total number of student-reported clinical activity types recorded as performed at least once across all 50 items than students at sites without trained CAPs (median 13.5, IQR 9-18, vs median 9, IQR 5-13; Mann-Whitney *U*=1850.5; *P*<.001; r=0.40, 95% CI 0.14-0.60).

### Weekly Trends in Student-Reported Clinical Activities

Weekly trends in the number of student-reported clinical activity types recorded as performed at least once across the 50 MCC-based activities are shown in Figure 3. The category × week interaction was not statistically significant in the Poisson GEE model (*P*=.45), and the Gaussian GEE sensitivity analysis yielded a similar result (*P*=.91). Thus, the temporal pattern of change did not show clear differences between the two site categories.

**Figure 3:**
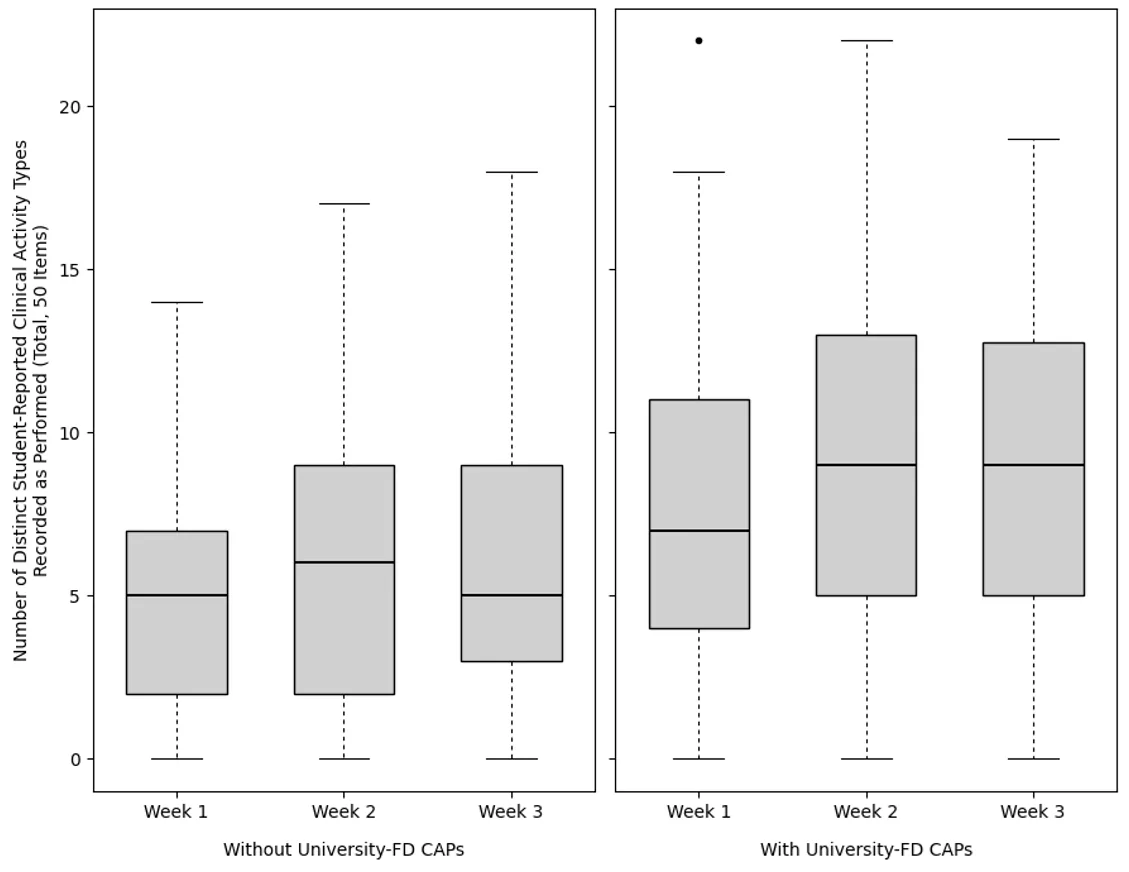
Weekly trends in student-reported clinical activity types recorded as performed at least once across all 50 items. Distinct activity types were defined as student-reported clinical activity types recorded as performed at least once during each clerkship week. Boxplots show distributions for students at sites with and without trained CAPs; horizontal lines indicate medians. The site category × week interaction was not statistically significant in the Poisson GEE model (*P*=.45). CAP: community-based attending physician; FD: faculty development; GEE: generalized estimating equation.

### Exploratory Sensitivity Analyses

The presence of trained CAPs and medically underserved placement highly overlapped in the observed data, leaving few discordant cases to reliably separate their independent associations. Of the 34 students at sites with trained CAPs, 33 (97.1%) were placed in medically underserved areas, whereas only 4 (5.1%) of 78 students at sites without trained CAPs were placed in such areas. This disparity precluded meaningful descriptive comparison of the sites outside medically underserved areas.

In the adjusted Poisson model with robust SEs, the association for the presence of trained CAPs was attenuated and not statistically significant after adjustment for home visit exposure, baseline rural self-efficacy, regional quota admission, sex, and site type (incidence rate ratio [IRR] 1.24, 95% CI 0.97-1.58; *P*=.09). Home visit exposure (IRR 1.47, 95% CI 1.08-2.01; *P*=.01) and baseline rural self-efficacy (IRR 1.17 per SD, 95% CI 1.03-1.32; *P*=.01) were associated with the primary outcome. In the contextual model with the “university FD program site” category replaced with “medically underserved placement,” the latter was associated with the primary outcome (IRR 1.31, 95% CI 1.02-1.68; *P*=.04). Models including both variables produced unstable estimates because of their limited overlap. Negative binomial sensitivity analyses showed a similar pattern. Detailed results are provided in Multimedia Appendix 6.

### Reflection Sheet Analysis of Clinical Skills

Table 2 presents data extracted from the reflection sheets. The analysis yielded 4097 textual units coded according to MCC learning objectives. Among the top 10 codes, procedure- and examination-related content appeared frequently, including treatment techniques (CS-03-02), physical findings (CS-01-02), investigation techniques (CS-03-01), and medical interviews (CS-01-01). Codes related to organ systems, including the digestive system (PS-02-08) and nervous system (PS-02-03), were less frequent among students at sites with trained CAPs.

In code-level comparisons, CS-03-02 was proportionally more frequent among students at sites without trained CAPs than among students at sites with trained CAPs (206/2780, 7.4%, vs 60/1317, 4.6%; *P*<.001). In contrast, CS-01-02, CS-03-01, and CS-01-01 did not show statistically significant differences between site categories. Codes related to organ systems, including PS-02-08 and PS-02-03, were proportionally more frequent at sites without trained CAPs (both *P*<.001). The clinical activity–related subtotal did not differ significantly between site categories.

**Table 2:** Deductive content analysis of students’ reflection sheets: top 10 codes.^a^

MCC^b^ code	Explanation	Without FD^c^-trained CAPs^d^ (n=2780), n (%)	With FD-trained CAPs (n=1317), n (%)	*P* value
CS-03-02^e^	Treatment techniques: implementation of appropriate patient care, including treatment	206 (7.4)	60 (4.6)	<.001
CS-01-02	Physical findings: gathering patient information	171 (6.2)	71 (5.4)	.34
IP-02-04^f^	Understanding of other professions: understanding the roles of other professions according to their work environment or affiliations	148 (5.3)	62 (4.7)	.40
PS-02-08^g^	Digestive system: normal structure and function of organs and systems, as well as pathogenesis, diagnosis, and treatment of diseases	173 (6.2)	33 (2.5)	<.001
PS-02-03	Nervous system: normal structure and function of organs and systems, as well as pathogenesis, diagnosis, and treatment of diseases	164 (5.9)	41 (3.1)	<.001
CS-03-01	Investigation techniques: implementation of appropriate patient care, including treatment	110 (4.0)	65 (4.9)	.15
CS-02-04	Treatment planning and progress evaluation: integration, analysis, and assessment of patient information and planning treatment	110 (4.0)	60 (4.6)	.37
PS-03-03	Infectious diseases: multisystemic physiological changes, as well as pathogenesis, diagnosis, and treatment of diseases affecting the whole body	93 (3.4)	54 (4.1)	.23
CS-01-01	Medical interviews: gathering patient information	84 (3.0)	55 (4.2)	.06
GE-02-04^h^	Primary care at home: community perspectives and approaches	78 (2.81)	28 (2.13)	.20
Clinical activity–related subtotal	Subtotal of CS-01-01, CS-01-02, CS-03-01, and CS-03-02	571 (20.54	251 (19.1)	.27

^a^Sites without trained CAPs (n=78) and sites with trained CAPs (n=34), where “n” denotes the total number of final human-verified MCC-coded textual units in each site category.

^b^MCC: Model Core Curriculum.

^c^FD: faculty development.

^d^CAP: community-based attending physician.

^e^CS: Clinical Skills.

^f^IP: Interprofessional Collaboration.

^g^PS: Problem-Solving.

^h^GE: Generalism.

### Representative Reflection Quotations

Representative quotations from students at sites with trained CAPs provided additional context regarding students’ reflections. One student described home care learning as an opportunity to understand patients’ living environments:

The strength of home visits is that by learning about the patient’s home and how the patient spends daily life, physicians can propose treatment plans that take the patient’s lifestyle into account.

Site with a trained CAP, week 1

In later reflections, the same student described more complex concerns about social context and patient values:

Although the patient was independent in activities of daily living, being older and living alone seemed dangerous because no one might notice if something happened. I felt that some intervention, other than monthly home visits, might be necessary. However, since the patient was satisfied with their life, I felt that adding an intervention might disrupt the patient’s way of life and values.

Site with a trained CAP, week 2

The student further noted:

Taking social vital signs (HEALTH-P) is very important for clarifying problems and determining the intervention for patients who live alone or have complex social issues.

Site with a trained CAP, week 3

Another student at a site with a trained CAP reflected on ethical decision-making and interprofessional care. After learning about Jonsen’s four-box method, the student wrote:

I learned that Jonsen’s four-box method is a necessary framework for decision-making that patients, families, and health care professionals can all accept.

Site with a trained CAP, week 2

After attending a multidisciplinary care conference at a patient’s home, the same student reflected:

In the care conference, it seemed difficult for the patient’s family to express their wishes in front of the patient. I could not find the correct answer for how we should elicit the family’s wishes.

Site with a trained CAP, week 2

The student further noted:

I learned that Jonsen’s four-box method is only a means of organizing information, not a method for creating a solution. I would like to consider how to use the organized information to find a solution.

Site with a trained CAP, week 2

Hence, students reflected on social determinants of health, patient values, ethical decision-making, interprofessional information sharing, and clinical reasoning in home care and community-based contexts.

### Integration of Digital Log and Reflection Sheet Findings

A joint display (Table 3) was constructed to integrate quantitative digital log findings with the deductively coded reflection sheet findings. The digital log analysis showed student-reported differences in activities across placement contexts, whereas the reflection sheet analysis provided complementary descriptive information on the learning content that students described. The integrated descriptive interpretation indicated that the two sources captured different dimensions of clerkship experience. The reflection sheet findings did not explain activity count differences, establish mechanisms, or validate the digital log counts.

**Table 3:** Joint display integrating quantitative digital log findings and deductively coded reflection sheet findings.

Quantitative digital log findings	Reflection sheet findings	Integrated descriptive interpretation
Students at sites with trained CAPs had more student-reported examination-based, lower-risk clinical activity types recorded as performed at least once.	Reflection sheets included procedure- and examination-related codes; however, the clinical activity–related subtotal did not differ significantly between site categories.	Logs and reflections captured different dimensions of learning; activity counts did not translate directly into reflection code density.
No statistically significant site category difference was observed for basic clinical and emergency procedures.	Reflections included safety discussions, patient context, home care, and decision-making.	Reflections provided complementary descriptive information and did not explain why activity counts differed.
Sensitivity analyses showed strong overlap between university FD program exposure and the placement context.	Quotations illustrated social determinants, ethics, interprofessional information sharing, and home care reasoning.	Observed differences are best interpreted as experiences within distinct placement contexts.

^a^CAP: community-based attending physician.

^b^FD: faculty development.

### Weekly Distribution of Procedure- and Examination-Related Reflection Codes

Table 4 summarizes the weekly distribution of code frequencies for clinical procedures and examinations. When the proportion of procedure- and examination-related codes was compared with the total number of codes in the reflection sheets, the highest proportion was observed in the first week, which decreased in the second and third weeks. Therefore, students’ reflections on procedure- and examination-related learning appeared most prominently early in the clerkship, whereas later reflections broadened toward other aspects of community-based clinical learning. For CS-01-02, the proportion was similar between site categories in week 1, lower at sites with trained CAPs in week 2, and directionally higher at those sites in week 3. However, this pattern was inconsistent and was not interpreted as evidence of progressive temporal change attributable to university FD program exposure.

**Table 4:** Weekly distribution of code frequencies for procedure- and examination-related practice.^a^

MCC^b^ code	Week 1			Week 2			Week 3	
	Without FD^c^-trained CAPs^d^ (n=815), n (%)	With FD-trained CAPs (n=424), n (%)	Without FD-trained CAPs (n=1000), n (%)		With FD-trained CAPs (n=459), n (%)	Without FD-trained CAPs (n=965), n (%)		With FD-trained CAPs (n=434), n (%)
CS-01-01^e^	35 (4.3)	23 (5.4)	23 (2.3)		13 (2.8)	26 (2.7)		16 (3.7)
CS-01-02	63 (7.7)	27 (6.4)	64 (6.4)		15 (3.3)	44 (4.6)		29 (6.7)
CS-03-01	35 (4.3)	20 (4.7)	38 (3.8)		23 (5.0)	37 (3.8)		22 (5.1)
CS-03-02	81 (9.9)	24 (5.7)	64 (6.4)		16 (3.5)	61 (6.3)		20 (4.6)
Code subtotal	214 (26.3)	94 (22.2)	189 (18.9)		67 (14.6)	168 (17.4)		87 (20.1)

^a^Sites without trained CAPs (n=78) and sites with trained CAPs (n=34), where “n” denotes the total number of final human-verified MCC-coded textual units within each site category and week. Weekly patterns are descriptive and should not be interpreted as progressive changes attributable to university FD program exposure.

^b^MCC: Model Core Curriculum.

^c^FD: faculty development.

^d^CAP: community-based attending physician.

^e^CS: Clinical Skills.

## Discussion

### Principal Findings

This formative evaluation demonstrated that the student-reported digital daily log workflow achieved 99.6% completion across eligible student-days and provided item- and week-level descriptions of clinical activity experiences across distributed sites. Students at sites with trained CAPs had more student-reported examination-based, lower-risk clinical activity types recorded as performed at least once, with the 50-item sensitivity analysis showing a similar pattern. However, placement category was closely intertwined with other contextual factors, and no statistically significant difference was observed for basic clinical and emergency procedures. Therefore, the findings do not establish an independent effect of supervision by trained CAPs.

#### Interpretation in the Placement Context

The observed differences were closely intertwined with the placement context. The recent literature on workplace-based learning in distributed health care settings emphasizes that clinical learning opportunities are shaped by local workflow, educator engagement, site readiness, and organizational culture [43]. This perspective is consistent with our finding that the presence of trained CAPs was strongly associated with medically underserved placement, home visit exposure, site readiness, local educational culture, and baseline rural self-efficacy. Exploratory sensitivity analyses suggested that the observed pattern was attenuated after adjustment for available contextual and student-level variables. Therefore, the findings highlight student-reported differences across distinct educational and clinical contexts. Accordingly, the two site categories should be interpreted as placement context categories rather than as intervention and control groups, and comparisons between them should not be interpreted as estimates of an independent effect of the university FD program.

Medically underserved and resource-limited clinical environments may promote student participation in clinical activities. In such settings, local patient care needs, staffing constraints, home visit exposure, care continuity, and integration of students into daily clinical workflow could create more opportunities for supervised student participation, independent of trained CAPs. Therefore, the observed differences reflect the broader clinical and educational context of medically underserved placements.

### Comparison With Prior Work and Educational Implications

Our findings should be interpreted in relation to previous work on clinical participation, community-based clerkships, and FD. Previous studies have reported gaps between medical students’ desired and actual procedural experiences before graduation [2,3]. Furthermore, community-based clerkships provide distinct learning opportunities through broader clinical exposure, care continuity, and direct patient care experiences [9]. In this study, students at sites with trained CAPs had a broader range of student-reported examination-based, lower-risk clinical activity types recorded as performed at least once; however, this pattern was closely intertwined with the placement context.

These findings further relate to the literature on FD in medical education. Reviews have suggested that FD can improve teaching effectiveness, support practice communities, and strengthen educational culture [14,15]. However, our study did not demonstrate the independent effectiveness of supervision by trained CAPs. Rather, the presence of such CAPs is part of a broader educational environment that includes medically underserved placement, home visit exposure, site readiness, local workflow, educator engagement, and local educational culture.

#### University FD Program Exposure as a Contextual Variable

The FD program included curriculum alignment, observation and feedback, workplace-based assessment, reflection, microteaching, coaching, and peer learning among CAPs. However, this study did not directly measure whether these components were implemented in day-to-day supervision or altered student participation. Hence, the presence of trained CAPs is a contextual characteristic. Future qualitative and longitudinal studies could examine how local structure, relationships, competing priorities, and shared educational values shape student participation across community-based settings [44,45].

#### Digital Education Data Contribution

Rather than demonstrating the independent causal effectiveness of FD, this study provides a digitally supported educational evaluation framework to describe how student-reported clinical activity experiences vary across community-based placements. Google Spreadsheet–based daily logs enabled students to record MCC-based clinical procedure and examination experiences at the item level across weeks, with substantive entries available for 99.6% of student-days. This digital structure allowed aggregation by activity type, week, and placement category. Previous works on e-logbooks showed that digital logging could support clinical exposure monitoring, skill development, and curriculum progression in decentralized clinical training settings [16]. However, the workflow was student reported and did not constitute supervisor-confirmed workplace assessment data. This supports our cautious interpretation that the observed differences were intertwined with placement context rather than attributable to supervision by trained CAPs alone. The human-verified AI-assisted preliminary sorting and candidate-coding workflow connects this study to emerging literature on Gen AI in medical education. Recent *JMIR Medical Education* studies have highlighted the educational potential of ChatGPT-based tools and the need for expert verification, evidence awareness, and safeguards [22,23]. In this study, detailed AI-human concordance was modest, and all final coding decisions were made by the researchers.

#### Interpretation of Reflection Sheet Findings

Although students at sites with trained CAPs had more examination-based, lower-risk clinical activities in the digital daily logs, this was not accompanied by a higher density of practice-related codes in the reflection sheets. However, this does not indicate less learning or weaker subjective meaning. The two data sources captured different dimensions of clerkship learning: the digital logs recorded student-reported clinical experiences, whereas the reflection sheets captured selective narratives of what students considered meaningful. Therefore, the proportion of practice-related codes is not a direct proxy for the amount or quality of learning. Students may have expressed their learning through reflections on communication, patient context, home care, interprofessional collaboration, professionalism, and community-based care rather than through procedure- or examination-related codes.

Direct quotations provided a complementary descriptive context regarding the relationship between digital log data and written reflections. Some reflections from sites with trained CAPs extended beyond procedures and examinations to broader competencies required for community-based care, including social determinants of health, patient values, advance care planning, ethical decision-making, interprofessional information sharing, and clinical reasoning under limited contextual and diagnostic resources. Hence, these quotations are illustrative descriptive information rather than evidence of a causal shift attributable to university FD program exposure.

#### Interpretation of Procedural Findings

The distinction between examination-based, lower-risk clinical activities and basic clinical or emergency procedures is important for interpreting these findings. Students at sites with trained CAPs had a wider range of student-reported examination-based, lower-risk clinical activity types recorded as performed at least once; however, a similar pattern was not observed for basic clinical or emergency procedures. Activities in the latter category were likely more constrained by clinical risk, patient safety, legal responsibility, institutional policy, and explicit patient consent. Since these factors were not directly measured, this interpretation remains descriptive.

The FD program was designed as a general educational development program for community-based physicians and did not provide procedure-specific entrustment criteria, standardized supervision protocols for individual procedures, or a uniform patient consent process. Future implementation of higher-risk student participation would require additional safeguards, including procedure-specific supervision guidelines, standardized entrustment criteria, explicit definitions of permissible student roles, structured documentation of supervisor oversight, and clear patient consent processes within each clinical site.

### Formative Implications for the Next Implementation Cycle

For the next implementation cycle, the digital log workflow should retain the practical daily structure that supported high completion, while prospectively storing completion, delayed-entry, reminder, and postreview correction indicators. Standardized orientation materials should explicitly document the operational definitions of “performed,” “observed/simulated,” and “not performed.”

Since universal item-level verification would impose substantial workload on community supervisors, selected clinical activity items could be confirmed prospectively. The digital log should be linked, where feasible, with CC-EPOC and other workplace-based assessment records to compare student-reported experiences with supervisor-confirmed performance, entrustment, and competency-related outcomes.

Student and supervisor interviews should examine usability, the reporting burden, reporting bias, and how local workflow influences documentation and participation. Future cohorts should include pre- and postimplementation data and better overlap between university FD program exposure and placement context. Furthermore, future research should assess whether performed clinical activities translate into competence, entrustment, professional identity formation, and retention in rural practice [46,47].

### Limitations

This study has several limitations. Students were not randomly assigned to clerkship sites, and the presence of trained CAPs was closely associated with medically underserved placement, home visit exposure, site readiness, local educational culture, and student background. Therefore, the study could not determine whether supervision by trained CAPs was independently associated with student-reported clinical activity experiences. Although we conducted exploratory sensitivity analyses, they could not eliminate the limited overlap between university FD program exposure and placement context. Table 1 includes site-level educational characteristics; however, unmeasured teaching-related exposure among supervisors, especially at sites without trained CAPs, may have affected the contrast between site categories.

The primary outcome was based on student-reported Google Spreadsheet logs. However, it was not independently confirmed by supervisors. Although faculty members monitored entries and the overall completion rate was high, the precise frequency and duration of delayed entries and the number of entries modified after faculty review were not prospectively stored as structured indicators. Furthermore, no standardized audit or partial supervisor confirmation procedure was implemented. CC-EPOC, Mini-CEX, EPA-related records, and other systematically collected workplace-based assessment data were unavailable for the 2024 off-campus cohort. Therefore, the outcome does not directly measure the actual number of times that procedures or examinations were performed, performance quality, procedural competence, entrustment level, or supervisor-confirmed performance. This reliance on student-entered logs may have introduced reporting or registration bias. Accordingly, future implementations should prospectively collect log process indicators and link selected student-reported activities with supervisor-confirmed workplace-based assessment or other educational assessment data.

The reflection sheet analysis used deductive MCC-based coding and was not designed to identify emergent qualitative themes or mechanisms. Moreover, weekly code-level patterns in reflection sheet data, including CS-01-02, were exploratory. Hence, important concepts outside the MCC framework may have been missed. Although all AI-generated candidate codes were reviewed by human researchers and final interrater agreement between the two human reviewers was excellent, the initial AI-human concordance was modest, particularly at the third-tier MCC code level. These findings indicate that Gen AI may be useful for preliminary sorting and consistency checking; however, granular deductive coding of reflective text cannot be delegated to Gen AI without human verification.

No formal a priori sample size calculation was performed because the study used a fixed cohort of eligible students from one academic year. Therefore, the study was not powered to estimate an independent effect of supervision by trained CAPs. Furthermore, the generalizability of this study is limited because it was conducted at a single university within a specific Japanese community-based clerkship system. Hence, the placement structure, FD program, regional quota admission system, medically underserved placement context, Japanese MCC framework, and locally developed Google Spreadsheet–based daily log workflow may differ from those of other institutions or countries. Therefore, the findings should be interpreted as exploratory evidence from one institution and region.

The evaluation period may not capture the long-term sustainability of the digital workflow. As the analysis focused on students’ reported experiences and reflection sheets, whether the observed differences translate into postclerkship performance, objective structured clinical examinations, workplace-based assessments, or later clinical competence remains unclear. The reflection sheet analysis was deductively mapped to MCC learning objectives and did not generate a comprehensive explanatory model of CAP teaching behaviors. Gen AI was used only to support preliminary sorting and candidate coding of deidentified textual units. The final coding decisions and interpretations were made by human researchers. Efficiency, time savings, workload reduction, and usability were not prospectively evaluated; therefore, no conclusions regarding efficiency or workload reduction can be drawn from this study. Hence, AI-generated candidate codes may introduce model-related suggestions or inconsistencies; we addressed this risk by necessitating human review and resolving uncertain cases through researcher discussion.

### Conclusion

This exploratory observational mixed methods study illustrated how a digital educational data workflow, combining student-reported daily clinical activity logs with human-verified AI-assisted reflection coding, can be used to describe clinical learning experiences across distributed community-based clerkship sites. The workflow yielded nearly complete longitudinal student-reported data and identified variation in student-reported clinical activity types recorded as performed at least once across placement contexts. However, these differences cannot be attributed independently to the presence of trained CAPs. Reflection sheets provided complementary descriptive information, and the AI component functioned as preliminary coding support that required human verification.

## Appendix

Multimedia Appendix 1 GRAMMS checklist. GRAMMS: Good Reporting of A Mixed Methods Study.

Multimedia Appendix 2 Structure and content of the university FD program for CAPs. CAP: community-based attending physician; FD: faculty development.

Multimedia Appendix 3 List of MCC-based clinical activities included in the digital daily log and author-defined analytic classification. MCC: Model Core Curriculum.

Multimedia Appendix 4 Conceptual diagram of contextual overlap and reporting processes relevant to student-reported clinical activity experiences.

Multimedia Appendix 5 Prompt templates, coding instructions, and representative outputs for the human-verified AI-assisted preliminary sorting and candidate-coding workflow. AI: artificial intelligence.

Multimedia Appendix 6 Exploratory sensitivity analyses for contextual confounding.

## Abbreviations

AI: artificial intelligence
CAP: community-based attending physician
CC-EPOC: Clinical Clerkship E-Portfolio of Clinical Training
CS: Clinical Skills
EPA: Entrustable Professional Activities
FD: faculty development
GEE: generalized estimating equation
Gen AI: generative artificial intelligence
GRAMMS: Good Reporting of A Mixed Methods Study
IP: Interprofessional Collaboration
IRR: incidence rate ratio
MCC: Model Core Curriculum
Mini-CEX: Mini-Clinical Evaluation Exercise
PS: Problem-Solving

## Data Availability

The datasets generated and analyzed during this study are not publicly available, because they contain information derived from students’ educational records and reflection sheets. Deidentified data may be available from the corresponding author upon reasonable request, subject to institutional approval and applicable ethical and privacy requirements.

## References

[1] Kim JY, Myung SJ (2014). Could clinical experience during clerkship enhance students' clinical performance?. BMC Med Educ.

[2] Dehmer JJ, Amos KD, Farrell TM, Meyer AA, Newton WP, Meyers MO (2013). Competence and confidence with basic procedural skills: the experience and opinions of fourth-year medical students at a single institution. Acad Med.

[3] Promes SB, Chudgar SM, Grochowski CO, Shayne P, Isenhour J, Glickman SW, Cairns CB (2009). Gaps in procedural experience and competency in medical school graduates. Acad Emerg Med.

[4] Button B, Gao M, Dabous J, Oandasan I, Bosco C, Cameron E (2023). The rural road map for action: an examination of undergraduate medical education in Canada. Can Med Educ J.

[5] Nishigori H (2024). Medical education in Japan. Med Teach.

[6] Japan Association of Medical Colleges (2025). Daigaku ni Okeru Iryojin Yosei no Arikata ni Kansuru Chosa Kenkyu Itaku Jigyo: Shinryo Sanka-gata Rinsho Jisshu no Juujitsu o Mokuteki to Shita Shidoi Yosei Program no Kaihatsu to Tenkai [Research project commissioned by MEXT on the Training of Medical Professionals in Universities: Development and Implementation of Faculty Development Programs Aimed at Enhancing Participation-Based Clinical Clerkships].

[7] Gruppen LD (2019). Context and complexity in the clinical learning environment. Med Teach.

[8] Chan M, Snell L, Philibert I (2019). The education avenue of the clinical learning environment: a pragmatic approach. Med Teach.

[9] Ohta R, Ryu Y, Katsube T, Otani J, Moriwaki Y (2021). Strengths and challenges for medical students and residents in rural Japan. Fam Med.

[10] Norris TE, Schaad DC, DeWitt D, Ogur B, Hunt DD, Consortium of Longitudinal Integrated Clerkships (2009). Longitudinal integrated clerkships for medical students: an innovation adopted by medical schools in Australia, Canada, South Africa, and the United States. Acad Med.

[11] Walters L, Greenhill J, Richards J, Ward H, Campbell N, Ash J, Schuwirth LWT (2012). Outcomes of longitudinal integrated clinical placements for students, clinicians and society. Med Educ.

[12] Thistlethwaite JE, Bartle E, Chong AAL, Dick M-L, King D, Mahoney S, Papinczak T, Tucker G (2013). A review of longitudinal community and hospital placements in medical education: BEME guide no. 26. Med Teach.

[13] Hudson JN, Poncelet AN, Weston KM, Bushnell JA, A Farmer E (2016). Longitudinal integrated clerkships. Med Teach.

[14] Steinert Y, Mann K, Anderson B, Barnett BM, Centeno A, Naismith L, Prideaux D, Spencer J, Tullo E, Viggiano T, Ward H, Dolmans D (2016). A systematic review of faculty development initiatives designed to enhance teaching effectiveness: a 10-year update: BEME guide no. 40. Med Teach.

[15] de Carvalho-Filho MA, Tio RA, Steinert Y (2019). Twelve tips for implementing a community of practice for faculty development. Med Teach.

[16] Barteit S, Schmidt J, Kakusa M, Syakantu G, Shanzi A, Ahmed Y, Malunga G, Blass K, Nieder J, Andreadis P, Neuhann F (2022). Electronic logbooks (e-logbooks) for the continuous assessment of medical licentiates and their medical skill development in the low-resource context of Zambia: a mixed-methods study. Front Med (Lausanne).

[17] Bijker R, Merkouris SS, Dowling NA, Rodda SN (2024). ChatGPT for automated qualitative research: content analysis. J Med Internet Res.

[18] Bowden J, Mohanakrishnan M, Thompson AR (2026). Incorporating artificial intelligence in qualitative research: exploring the role of ChatGPT in thematic analysis. Med Sci Educ.

[19] Yue Y, Liu D, Lv Y, Hao J, Cui P (2025). A practical guide and assessment on using ChatGPT to conduct grounded theory: tutorial. J Med Internet Res.

[20] Kondo T, Miyachi J, Jönsson A, Nishigori H (2024). A mixed-methods study comparing human-led and ChatGPT-driven qualitative analysis in medical education research. Nagoya J Med Sci.

[21] Luo X, Tham YC, Giuffrè M, Ranisch R, Daher M, Lam K, Eriksen AV, Hsu C, Ozaki A, Moraes FYD, Khanna S, Su K, Begagić E, Bian Z, Chen Y, Estill J, GAMER Working Group (2025). Reporting guideline for the use of generative artificial intelligence tools in medical research: the GAMER statement. BMJ Evid Based Med.

[22] Tangadulrat P, Sono S, Tangtrakulwanich B (2023). Using ChatGPT for clinical practice and medical education: cross-sectional survey of medical students’ and physicians’ perceptions. JMIR Med Educ.

[23] Ebihara H, Kasai H, Shimizu I, Shikino K, Tajima H, Kimura Y, Ito S (2025). Development of a clinical clerkship mentor using generative AI and evaluation of its effectiveness in a medical student trial compared to student mentors: 2-part comparative study. JMIR Med Educ.

[24] Allemang B, Sitter K, Dimitropoulos G (2022). Pragmatism as a paradigm for patient-oriented research. Health Expect.

[25] Brown MEL, Dueñas AN (2020). A medical science educator’s guide to selecting a research paradigm: building a basis for better research. Med Sci Educ.

[26] Elo S, Kyngäs H (2008). The qualitative content analysis process. J Adv Nurs.

[27] Guetterman TC, Fetters MD, Creswell JW (2015). Integrating quantitative and qualitative results in health science mixed methods research through joint displays. Ann Fam Med.

[28] O'Cathain A, Murphy E, Nicholl J (2008). The quality of mixed methods studies in health services research. J Health Serv Res Policy.

[29] Mazurkiewicz R, Friedman E, Karani R, Lin JJ (2013). Expectations for medical student work hours in inpatient clinical clerkships. Teach Learn Med.

[30] Weinstein A, MacPherson P, Schmidt S, Van Opstal E, Chou E, Pogemiller M, Gibbs K, Held M (2023). Needs assessment for enhancing pediatric clerkship readiness. BMC Med Educ.

[31] Alexandraki I, Kern A, Beck Dallaghan GL, Baker R, Seegmiller J (2024). Motivators and barriers for rural community preceptors in teaching: a qualitative study. Med Educ.

[32] Alexandraki I, Baker R, Kern A, Beck Dallaghan GL, Seegmiller J (2023). Faculty development for community preceptors: a narrative review of the literature. J Gen Intern Med.

[33] Nomura O, Komatsu H, Matsuyama Y, Onoue T, Ikusaka M, Okazaki H, Konishi Y (2024). Development of medical knowledge content for problem-solving competencies through dialogue with the undergraduate medical education community in Japan. Med Teach.

[34] Medical Education Model Core Curriculum Coordination Committee, Medical Education Model Core Curriculum Expert Research Committee (2023). Model core curriculum for medical education: AY 2022 revision. Ministry of Education, Culture, Sports, Science and Technology (MEXT), Japan.

[35] Hennus MP, Jarrett JB, Taylor DR, ten Cate O (2023). Twelve tips to develop entrustable professional activities. Med Teach.

[36] ten Cate TO, Snell L, Carraccio C (2010). Medical competence: the interplay between individual ability and the health care environment. Med Teach.

[37] Kawamoto R, Ninomiya D, Akase T, Uemoto A, Kumagi T (2018). Rural self-efficacy measuring intent for rural practice among Japanese medical students. Rural Remote Health.

[38] Takayama A, Poudyal H (2021). Incorporating medical supply and demand into the index of physician maldistribution improves the sensitivity to healthcare outcomes. J Clin Med.

[39] Shikino K, Nishizaki Y, Kataoka K, Nojima M, Shimizu T, Yamamoto Y, Fukui S, Nagasaki K, Yokokawa D, Kobayashi H, Tokuda Y (2024). Association between physicians' maldistribution and core clinical competency of resident physicians: a nationwide cross-sectional study. BMJ Open.

[40] Singh M, Lawrence R, Headrick L (2011). Expanding educators' medical curriculum tool chest: minute papers as an underutilized option for obtaining immediate feedback. J Grad Med Educ.

[41] Urushibara-Miyachi Y, Matsushita K, Nishigori H (2024). Development of a competency-based national model core curriculum in Japan: a case study. Med Teach.

[42] In J, Lee DK (2024). Alternatives to the *P* value: connotations of significance. Korean J Anesthesiol.

[43] Verhees MJM, Landstra AM, Engbers R, Koksma JJ, Laan RFJM (2024). Exploring workplace-based learning in distributed healthcare settings: a qualitative study. BMC Med Educ.

[44] Jolly B, Steinert Y (2025). Faculty development for organizational change. Faculty Development in the Health Professions: A Focus on Research and Practice. 2nd ed. Vol 11.

[45] Bolman LG, Deal TE (1984). Modern Approaches to Understanding and Managing Organizations. Jossey-Bass.

[46] Klumpp S, Rocha I, Amirfazli C, Bear C, DeYoung J, Gudi M, Patel M, DeWeert D (2025). Impact of early procedural exposure on pre-clinical medical students’ confidence. Cureus.

[47] Foster KE, Shah D, Plunkett Q, Sifri R (2024). Understanding faculty perspectives on health equity curriculum implementation in graduate medical education: a qualitative study. BMC Med Educ.

