# Supplementary material for: Using Student-Reported Digital Daily Logs and Human-Verified AI-Assisted Reflection Coding to Describe Clinical Experiences in Community-Based Clerkships: Exploratory Observational Mixed Methods Study
Source: JMIR Form Res. 2026 Sep 29;10:e102390. doi: 10.2196/102390 (PMC13662440; doi:10.2196/102390)
Supplement: Supplementary file 1 [file formative_v10i1e102390_app1.docx]

**Good Reporting of A Mixed Methods Study (GRAMMS) Checklist**

**Reporting guideline:**

Good Reporting of A Mixed Methods Study (GRAMMS) [1]

| **GRAMMS item** | **Reported?** | **Location in manuscript** | **How the item is addressed** |
| --- | --- | --- | --- |
| **Describe the justification for using a mixed-methods approach for the research question.** | Yes | Introduction, Methods: Study Design | The manuscript explains that distributed community-based clerkships provide diverse clinical learning opportunities. However, monitoring day-to-day clinical activity experiences across multiple sites remains challenging. Quantitative digital daily logs describe student-reported clinical activity experiences at item and week levels. Deductive content analysis of reflection sheets provides complementary descriptive information about the learning content students chose to document. |
| **Describe the design in terms of the purpose, priority, and sequence of methods.** | Yes | Methods: Study Design | The manuscript identifies the study as an exploratory observational mixed-methods study, incorporating quantitative digital log analysis and deductive content analysis of reflection sheets. The quantitative component is primary and describes activity experiences while exploring variation across placement contexts. The reflection-sheet analysis provides complementary descriptive context rather than a mechanistic explanation. Integration occurs through a joint display with an Integrated Descriptive Interpretation column. |
| **Describe each method in terms of sampling, data collection, and analysis.** | Yes | Methods: Setting and Participants, Quantitative Data Collection and Analysis, Implementation and Quality Control of the Digital Daily Log Workflow, Qualitative Data Collection and Analysis, Human-Verified AI-Assisted Preliminary Sorting and Candidate-Coding Workflow, Joint Display | The sampling frame comprised 112 eligible fifth-year medical students. Quantitative data were collected through Google Spreadsheet-based daily logs of 50 MCC-based clinical activities. The primary outcome was the number of student-reported examination-based, lower-risk clinical activity types recorded as performed at least once. Implementation quality was assessed across 1497 eligible student-days, and a classification-independent analysis examined all 50 items. Reflection sheets were collected on eligible scheduled clerkship days and deductively coded according to MCC learning objectives. A human-verified AI-assisted preliminary sorting and candidate-coding workflow supported coding. Researchers made the final decisions. |
| **Describe where integration has occurred, how it has occurred, and who has participated in it.** | Yes | Methods: Study Design, Methods: Joint Display, Results: Integration of Digital Log and Reflection-Sheet Findings, Table 3, Discussion: Interpretation of Reflection-Sheet Findings | Integration occurred through a joint display at the interpretation stage. The joint display aligned quantitative findings with corresponding MCC-coded reflection-sheet findings and integrated descriptive interpretations. The research team conducted the integration during analysis and manuscript development. Integration described convergence and divergence between the data sources. |
| **Describe any limitation of one method associated with the presence of the other method.** | Yes | Methods: Deductive Content Analysis, Methods: Human-Verified AI-Assisted Preliminary Sorting and Candidate-Coding Workflow, Methods: Joint Display, Discussion: Interpretation of Reflection-Sheet Findings, Limitations | The digital daily logs captured student-reported clinical activity experiences. However, they did not measure repetitions, quality, competence, entrustment, or supervisor-confirmed performance. The reflection-sheet analysis was deductive and was not designed to generate emergent themes or infer teaching mechanisms. Therefore, integration provided complementary descriptive context, yet could not validate either data source. AI-generated candidate codes required human verification. |
| **Describe any insights gained from mixing or integrating methods.** | Yes | Discussion: Interpretation of Digital Log and Reflection-Sheet Findings, Table 3, Discussion: Digital Educational Data Contribution, Discussion: Interpretation of Reflection-Sheet Findings, Limitations | The integrated interpretation showed that the digital logs and reflection sheets captured different dimensions of clerkship learning. Students at sites with CAPs trained in the university FD program had more student-reported examination-based, lower-risk clinical activity types recorded as performed at least once. However, this was not accompanied by a higher clinical activity-related reflection-code subtotal. Reflections included safety, patient context, home care, ethics, interprofessional information sharing, and decision-making. The combined findings supported a descriptive, contextual interpretation. |

## Reference

O’Cathain A, Murphy E, Nicholl J. The quality of mixed methods studies in health services research. *J Health Serv Res Policy*. 2008;13(2):92-98. doi:10.1258/jhsrp.2007.007074
