# Supplementary material for: Using Student-Reported Digital Daily Logs and Human-Verified AI-Assisted Reflection Coding to Describe Clinical Experiences in Community-Based Clerkships: Exploratory Observational Mixed Methods Study
Source: JMIR Form Res. 2026 Sep 29;10:e102390. doi: 10.2196/102390 (PMC13662440; doi:10.2196/102390)
Supplement: Supplementary file 2 [file formative_v10i1e102390_app2.docx]

**Structure and content of the university faculty development (FD) program for community-based attending physicians (CAPs).**

The FD program consisted of weekly 2-hour sessions delivered primarily online, with in-person sessions held approximately once per month. Across the 38 available session agendas reviewed for this study, this corresponded with approximately 76 scheduled contact hours. Participation was mandatory for enrolled CAPs, and the overall attendance rate was 99.3% on a person-session basis. Delivery fidelity was monitored through session agendas, attendance records, documentation of session outputs, and self-assessment and reflection forms. However, the extent to which each FD component was implemented in daily clinical supervision at each site was not directly observed or quantitatively assessed.

| **Content domain (category)** | **Illustrative topics included in the agenda** | **Delivery formats** | **Outputs** | **Frequency, n sessions** |
| --- | --- | --- | --- | --- |
| Program orientation & community building | Introductions; overview of community settings; program logistics | Brief lecture; sharing | Shared understanding of roles and participation approach | 2 |
| Competency framework for CAPs | Defining CAP competencies (e.g., affinity diagram); competency self-assessment; annual reflection | Group work; discussion; form-based reflection | Competency framework drafts; self-assessment entries | 4 |
| Curriculum design & alignment | Backward design; syllabus; alignment with MCC/outcomes; site-specific goals for year 1, year 5, and summer placements; student matching and placement planning | Lecture; planning workshops; peer discussion | Site-specific goals mapped to MCC/outcomes; placement plans and schedules | 17 |
| Teaching & learning methods | Work-based learning; coaching; observation and feedback; PBL/TBL; teaching in outpatient/home-care contexts | Lecture; peer sharing | Practical teaching and coaching strategies for outpatient and home-care contexts | 2 |
| Learner assessment & workplace-based assessment (WBA) | Formative/summative assessment; observation tools; WBL/WBA; workplace-based assessment in clinical settings | Lecture; practice/sharing | Assessment approaches and tool use plans | 4 |
| Microteaching & feedback | Microteaching methods and practice; video-based self-feedback and group feedback | Microteaching; feedback session | Microteaching delivery and feedback notes | 3 |
| Simulation-based education | Simulation planning and implementation; debriefing; community-oriented simulation sessions; reflection and iterative improvement | Planning workshops; implementation; observation; reflection | Simulation plans/materials; debriefing approach; revised scenarios | 8 |
| Program evaluation & educational analytics (IR) | Program/course evaluation; IR; using prior evaluation results to inform improvements | Lecture; discussion | Improvement ideas informed by evaluation data | 3 |
| Mentoring & learner support | Mentoring roles; mentor system; coordination for regional quota student mentoring | Lecture; coordination/planning | Mentoring plans and coordination outputs | 3 |
| Managing challenging situations | Difficult learner encounters | Lecture; discussion | Shared approaches to difficult learner situations | 1 |
| Interprofessional education (IPE) | Interprofessional collaboration capabilities; teaching IPE in community settings | Discussion; planning workshops | IPE-oriented teaching plans | 2 |
| Educational technology | Use of ICT/AV systems for teaching and learning | Demonstration/sharing | Plans for ICT/AV-supported teaching | 1 |
| Site visits & peer learning | Site visit rehearsal, participation, reporting, and reflection; peer-learning exchanges | Site visits; rehearsal/reporting; reflection | Site visit reports; peer-learning takeaways | 9 |
| Well-being & mental health | Physician mental health; teaching-related stress and buffering factors; workshops | Lecture; workshop | Shared stress-mitigation ideas | 3 |
| Career/policy & stakeholder engagement | Prefecture career program; scholarships/policy; stakeholder input surveys; collaboration with local government | Lecture; survey planning; briefing | Stakeholder survey planning/outputs; policy and career insights | 4 |
| Scholarship & dissemination | Educational practice research; conference rehearsal; abstract/idea consultation; progress updates | Rehearsal; consultation; reporting | Refined abstracts; study plans and deliverables | 5 |
| International collaboration / projects | Leicester/Inje collaborations; site-visit exchanges; student reception; project updates | Briefing; rehearsal/reporting | Project progress documentation and reports | 6 |

**Note.** Frequencies represent the number of sessions in which each topic appeared based on 38 reviewed session agendas. Since several sessions included more than one topic, frequencies across categories are not mutually exclusive and do not sum to 38. MCC = Model Core Curriculum for Medical Education in Japan; WBA = workplace-based assessment; WBL = workplace-based learning; IR = institutional research.
