# Supplementary material for: Using Student-Reported Digital Daily Logs and Human-Verified AI-Assisted Reflection Coding to Describe Clinical Experiences in Community-Based Clerkships: Exploratory Observational Mixed Methods Study
Source: JMIR Form Res. 2026 Sep 29;10:e102390. doi: 10.2196/102390 (PMC13662440; doi:10.2196/102390)
Supplement: Supplementary file 3 [file formative_v10i1e102390_app3.docx]

| **List of Model Core Curriculum (MCC)-based clinical activities included in the digital daily log and author-defined analytic classification** | |
| --- | --- |
| **Examination-based and lower-risk clinical activities (23 items)** | **Basic clinical procedures and emergency procedures (27 items)** |
| Medical interview | Skin antisepsis |
| Check the vital signs (body temperature, heart rate, blood pressure, respiratory rate, oxygen saturation) | Application of topical medications |
| Examination of head (face, hair, scalp, and skull), eye (visual field, pupil, light reflex, eye movement, proptosis, conjunctiva), ear (assessment of the pinna and hearing) | Airway suction |
| Examination of the external auditory canal and eardrum using an otoscope | Nebulizer |
| Examination of the lips, oral cavity, pharynx, and tonsils | Venous blood sampling |
| Examination of the sinuses | Peripheral venous catheterization |
| Observation of the anterior nasal cavity using a rhinoscope  Examination of thyroid gland, cervical vessels, trachea, and salivary glands | Insertion and extraction of nasogastric tube |
| Examination of the lymph nodes of the head and neck | Insertion and extraction of urinary catheter |
| Inspection, palpation, and percussion of the chest | Intradermal injection |
| Auscultation of breath sounds and added sounds | Subcutaneous injection |
| Auscultation of heart sounds and murmurs, Inspection, auscultation (bowel sounds and bruits), percussion, and palpation of the abdomen  Identification of pain on percussion over the spine | Intramuscular injection |
| Digital rectal examination (including prostate examination) | Intravenous injection |
| Breast examination | Urinalysis (including pregnancy test) |
| Assessment of level of consciousness | Microbiological testing (including gram staining) |
| Examination of the cranial nerves | Recording of a 12-lead electrocardiogram (ECG) |
| Examination of the fundi | Rapid bedside ultrasound (including Focused Assessment with Sonography for Trauma (FAST) for clinical decision-making) |
| Examination of tendon reflexes | Rapid antigen/pathogen testing |
| Examination of cerebellar function and the motor system  Examination of the sensory system (pain, temperature, touch, and deep sensation) | Blood glucose test |
| Identification of signs of meningeal irritation | Aseptic technique |
| Examination of limbs and spine (kyphosis and pain)  Examination of joints (range of motion, swelling, pain, and deformity)  Musculoskeletal examination (manual muscle strength testing)  Gynecological examination (bimanual examination and use of Cusco’s speculum) | Surgical hand washing |
| Comprehensive geriatric assessment (CGA) | Gowning techniques in the operating room |
| Medical record documentation | Basic sutures and suture removal |
| Repositioning and patient transfer | Basic life support (BLS) |
|  | Airway management |
|  | Chest compressions |
|  | Ventilation using bag-valve mask |
|  | Automated external defibrillator (AED) |
| Note: The 50 clinical activities were selected based on the Model Core Curriculum for Medical Education in Japan: 2022 Revision and the Guidelines for Participatory Clinical Clerkship. For analysis, the activities were grouped into two author-defined categories: examination-based, lower-risk clinical activities, and basic clinical and emergency procedures. This classification was developed for the present exploratory analysis, was hypothesis-generating, and was neither an official MCC classification nor an externally validated taxonomy. Activities within each category may differ in invasiveness, sensitivity, patient-consent requirements, supervision needs, and feasibility in community-based settings. A classification-independent sensitivity analysis was additionally conducted across all 50 items. Some closely related physical examination items from the Model Core Curriculum were integrated for daily log recording and analysis. Position change and patient transfer were included in the examination-based and lower-risk clinical activity category because they were considered non-invasive patient care and functional assessment activities in community-based clerkship settings. MCC = Model Core Curriculum for Medical Education in Japan. | |
