# Supplementary material for: Using Student-Reported Digital Daily Logs and Human-Verified AI-Assisted Reflection Coding to Describe Clinical Experiences in Community-Based Clerkships: Exploratory Observational Mixed Methods Study
Source: JMIR Form Res. 2026 Sep 29;10:e102390. doi: 10.2196/102390 (PMC13662440; doi:10.2196/102390)
Supplement: Supplementary file 4 [file formative_v10i1e102390_app5.docx]

**Prompt templates, coding instructions, and representative outputs for the human-verified AI-assisted preliminary sorting and candidate-coding workflow [1-3]**

**1. Purpose of AI Use**

Generative artificial intelligence (GenAI) supported preliminary sorting and generation of candidate Model Core Curriculum codes from medical students’ daily reflection sheets. A custom GPT was created using ChatGPT (GPT-4o; OpenAI) to map de-identified textual units from students’ reflection sheets with the codes in the Japanese Model Core Curriculum for Medical Education.

AI use supported initial organization and candidate coding of textual units. The AI tool suggested candidate Model Core Curriculum codes, provided brief rationales, and indicated whether suggested codes existed in the uploaded reference material. AI output was used as preliminary support; all final coding decisions were checked and confirmed by the researchers.

No fine-tuning was performed. Since the tool used the ChatGPT custom interface, model parameters, such as temperature and top-p, were not manually specified or recorded. Reporting of the human-verified AI-assisted workflow was guided by the GAMER framework, including tool specification, prompting approach, role of the AI tool, human verification, data privacy, and impact on conclusions.

**2. Data Protection and Deidentification**

Before the AI-assisted preliminary sorting and candidate-coding step, students’ reflection sheets were deidentified and divided into textual units. Identifying information, including student names, student identification numbers, patient names, clinical site names, and other personal identifiers, was removed before the textual units were processed. The custom GPT was configured in advance using the official Japanese Model Core Curriculum (MCC) for Medical Education published by the Ministry of Education, Culture, Sports, Science and Technology as a reference document. The PDF was uploaded as source material for the custom GPT before coding. Only deidentified textual units and coding instructions were entered into the AI tool. AI-generated candidate codes were reviewed by human researchers and were not treated as final coding.

**3. Reference Material and Coding Framework Provided to the Custom GPT**

The Japanese MCC for Medical Education is organized into four hierarchical tiers. The first tier comprises 10 broad competency domains; the second tier comprises 37 competency areas nested within those domains; the third tier comprises 125 specific learning categories; and the fourth tier comprises 595 detailed learning objectives nested within the third-tier categories. The custom GPT was instructed to classify input text according to this hierarchy. The first-tier competency domains included the following categories:

| **First-Tier Code** | **Domain** |
| --- | --- |
| PR | Professionalism |
| GE | Generalism |
| LL | Lifelong learning |
| RE | Research |
| PS | Problem-solving ability based on professional knowledge |
| IT | Information technology |
| CS | Clinical skills and patient care |
| CM | Communication |
| IP | Interprofessional collaboration |
| SO | Medicine in society |

The custom GPT was instructed to first identify 1 to 3 relevant first-tier domains, briefly explain the rationale, select the most relevant domain, and map the textual unit to lower-tier MCC codes whenever possible. The GPT was instructed to identify fourth-tier codes when the context allowed. When the textual unit was too abstract to identify a fourth-tier code, the GPT was instructed to stop at the third tier and explain the reason. In the final human-verified analytic dataset, coding was standardized at the third-tier. Although the GPT could suggest fourth-tier candidates, fourth-tier codes were not used as the analysis unit or included in the reported concordance analyses. AI–human concordance was evaluated at the first-, second-, and third-tier. Frequency summaries and weekly site-category comparisons in the main study were conducted at the third-tier. The GPT was further instructed to verify that each output code existed in the uploaded reference material and to avoid generating nonexistent codes. The output format included a code existence check mark.

**4. Initial Custom GPT Instruction**

The following instruction was used to configure the custom GPT. The original prompt was written in Japanese; an English translation is provided here for reporting purposes. This GPT supports mapping of sentences, paragraphs, or file contents to fourth-tier codes based on the Japanese MCC for Medical Education.

When the user inputs a sentence or text, identify 1 to 3 relevant first-tier competency domains, such as PR, GE, LL, RE, PS, IT, CS, CM, IP, or SO, and briefly explain the rationale for each. Do not wait for the user to select a category. Select the most relevant major domain and display the corresponding second-, third-, and fourth-tier codes.

When classifying text, do not overclassify a sentence into GE-03-06 (end-of-life care) merely because it contains negative expressions, such as “could not,” “refused,” or “was difficult.” Consider GE-03-06 only when end-of-life-related keywords, such as end-of-life care, advance care planning, or dignified death, are explicitly included or clearly indicated by the context.

Specify the fourth-tier code whenever possible. If the context is abstract and cannot be classified to the fourth tier, stop at the third tier and explain the reason. If classification is uncertain, present multiple fourth-tier candidates in order of relevance.

All output codes must be strictly checked against the uploaded latest code list or source material. Present only codes that exist in the uploaded material. Do not infer or generate nonexistent codes. If no exact code exists, clearly state that the code does not exist and provide the closest existing code as supplementary information.

When mapping codes based on the uploaded Model Core Curriculum PDF, apply the same two-step structure: first identify the broad competency domain and then provide detailed lower-tier codes.

For PS classification, carefully distinguish PS-01, PS-02, and PS-03:
- PS-01: Basic medical sciences, including cells, metabolism, structure, anatomy, physiology, mechanisms of immune response, causes of disease, and biological reactions.
- PS-02: Organ- or disease-based content, including specific organs, diseases, diagnosis, treatment, and treatment strategies.
- PS-03: Systemic or cross-cutting themes, including infectious diseases, oncology, emergency medicine, intensive care, genomic medicine, radiation medicine, lifelong learning, or interdisciplinary clinical issues.

For third-tier PS classification:
- If a disease name is described together with diagnosis or treatment, consider PS-02-XX.
- If infection, immunity, oncology, genomics, emergency medicine, or systemic/cross-organ issues are described, consider PS-03-XX.
- If terms such as reaction, structure, metabolism, inflammatory response, physiological change, or etiology are used, consider PS-01-XX.
- Even when the context is not explicit, infer possible codes from symptoms or procedures and present multiple candidates when appropriate.

Rules for drug-related content:
- If the context concerns selecting, administering, or evaluating medication as a treatment strategy, classify it as CS-02-04, treatment planning and progress evaluation.
- If the context concerns pharmacological action, pharmacokinetics, mechanisms of adverse effects, or basic pharmacology, classify it under PS-01-XX.
- If the context concerns pharmacotherapy for a specific organ-based disease, classify it under PS-02-XX.
- If the context concerns cross-cutting topics such as cancer chemotherapy, selection of antimicrobial agents, or systemic management of immunosuppressants, classify it under PS-03-XX.

Rules for LL classification:
- When the content is classified under LL, lifelong learning, also list other potentially relevant codes, such as PR, RE, or IT.
- Avoid classifying content only as LL. Consider whether the content also relates to other competency domains.

When interpreting the text, even if ambiguity remains, present the most similar or relevant codes where possible. Use clear and polite Japanese suitable for educators and health care professionals. Prioritize accuracy and logical mapping based on the code hierarchy.

Output the results in table format and include code existence verification using check marks or cross marks.

**5. Batch Coding Procedure**

The deidentified textual units were entered into the custom GPT in batches of approximately 20 units. The GPT generated a table with the following columns:

| **Column** | **Description** |
| --- | --- |
| # | Sequential number of the textual unit in the batch |
| Description | Deidentified textual unit from the reflection sheet |
| First-tier domain | First-tier Model Core Curriculum domain selected by the GPT |
| Code | Candidate lower-tier Model Core Curriculum code |
| Existence check | Check mark indicating whether the code existed in the uploaded reference material |
| Supplementary note | Brief rationale or note explaining the classification |

The typical output format was as follows:

| **#** | **Description** | **First-tier domain** | **Code** | **Existence check** | **Supplementary note** |
| --- | --- | --- | --- | --- | --- |
| 1 | [Deidentified textual unit] | IP | IP-01-01 | ✅ | Brief rationale |
| 2 | [Deidentified textual unit] | CS | CS-01-02 | ✅ | Brief rationale |
| 3 | [Deidentified textual unit] | GE | GE-02-04 | ✅ | Brief rationale |

The output was reviewed by the researchers. When a code was inappropriate, the researchers provided a corrective instruction and asked the GPT to reclassify the relevant batch or subset.

**6. Corrective Prompts Used During Coding**

During the coding process, the research team observed that some classifications, especially lower-tier classifications within the PS category, were inappropriate. When this occurred, the researchers provided specific corrective instructions. An example of a corrective prompt is shown below. The original prompt was written in Japanese; an English translation is provided here.

Some classifications under PS-03 may be incorrect. Please carefully check the lower-tier classifications and reclassify them.

Confirm whether each textual unit should be classified under PS-01, PS-02, or PS-03. Then check the appropriate third- and fourth-tier codes.

Do not classify content under PS-03 only because it contains general clinical or disease-related wording. If the content concerns a specific organ system or disease, consider PS-02. If it concerns basic mechanisms, structure, metabolism, biological reactions, or pathophysiology, consider PS-01. If it concerns infection, oncology, emergency medicine, intensive care, genomic medicine, radiation medicine, or a systemic/cross-cutting theme, consider PS-03.

Please provide corrected candidate codes in table format and briefly explain the reason for each correction.

Other corrective instructions were given when the GPT overclassified content based on keywords rather than context. For example, the researchers instructed the GPT not to classify negative expressions, such as “could not” or “was difficult,” as end-of-life care unless the text explicitly concerned end-of-life care, advance care planning, dignified death, or a related context.

**7. Representative AI Output**

The following table shows a representative output from the custom GPT. The original output was in Japanese. An English translation is provided for reporting purposes. The table illustrates the actual output format used during coding.

| **#** | **Description** | **First-tier domain** | **Code** | **Existence check** | **Supplementary note** |
| --- | --- | --- | --- | --- | --- |
| 1 | About the work of pharmacists. | IP | IP-01-01 | ✅ | Understanding the work of another profession, specifically pharmacists. |
| 2 | I was able to understand the work content, but I could not contribute in practice. | IP | IP-01-01 | ✅ | Challenge related to understanding professional roles and participating in practice. |
| 3 | I would like to learn more deeply about the actual work of other professions. | IP | IP-01-01 | ✅ | Desire to further understand other professions; overlaps with interprofessional understanding. |
| 4 | How junior high school students today think and feel. | CM | CM-03-01 | ✅ | Understanding local residents, especially adolescents. |

The full batch output contained approximately 20 textual units per run. The GPT output was reviewed by the researchers, and final codes were confirmed or corrected manually.

**8. Representative Human Review Decisions**

The following examples illustrate how AI-generated coding suggestions were handled by the researchers. These examples show the review process rather than presenting additional study findings.

**8.1 Accepted AI Suggestion**

| **Item** | **Content** |
| --- | --- |
| Deidentified textual unit | About the work of pharmacists. |
| AI-suggested first-tier domain | IP |
| AI-suggested code | IP-01-01 |
| Existence check | ✅ |
| AI supplementary note | Understanding the work of another profession, specifically pharmacists. |
| Human decision | Accepted |
| Final code | IP-01-01 |
| Reason | The textual unit directly concerned understanding the work of pharmacists as another profession. |

**8.2 Accepted AI Suggestion**

| **Item** | **Content** |
| --- | --- |
| Deidentified textual unit | I would like to learn more deeply about the actual work of other professions. |
| AI-suggested first-tier domain | IP |
| AI-suggested code | IP-01-01 |
| Existence check | ✅ |
| AI supplementary note | Desire to further understand other professions. |
| Human decision | Accepted |
| Final code | IP-01-01 |
| Reason | The main learning content was interprofessional understanding. |

**8.3 Revised AI Suggestion**

| **Item** | **Content** |
| --- | --- |
| Deidentified textual unit | I learned how the physician selected medication based on symptoms, test results, and the patient’s living situation. |
| Initial AI-suggested code | PS-01-related code |
| Researcher review | The text did not concern pharmacological mechanisms or basic pharmacology. |
| Final code | CS-02-04 |
| Reason | The main content was medication selection as part of treatment planning and progress evaluation. |

**8.4 Corrected PS Classification**

| **Item** | **Content** |
| --- | --- |
| Deidentified textual unit | I learned how symptoms and examination findings are used to consider neurological disease and decide further evaluation. |
| Initial AI-suggested category | PS-03 |
| Corrective prompt | Some classifications under PS-03 may be incorrect. Please carefully check the lower-tier classifications. |
| Revised AI-suggested code | PS-02-03 |
| Human decision | Accepted after review |
| Final code | PS-02-03 |
| Reason | The unit concerned a specific organ system, the nervous system, rather than a systemic or cross-cutting PS-03 topic. |

**8.5 Ambiguous Case Requiring Human Review**

| **Item** | **Content** |
| --- | --- |
| Deidentified textual unit | I was able to understand the work content, but I could not contribute in practice. |
| AI-suggested first-tier domain | IP |
| AI-suggested code | IP-01-01 |
| Existence check | ✅ |
| Human review | Required |
| Final code | IP-01-01 |
| Reason | Although the student described difficulty contributing in practice, the main content was understanding another profession’s work. The negative expression did not indicate end-of-life care or another unrelated category. |

**9. Human Review Process**

The AI-assisted coding process followed these steps:

1. Students’ reflection sheets were deidentified
2. The deidentified reflection sheets were divided into textual units.
3. A custom GPT was created using ChatGPT (GPT-4o; OpenAI).
4. The official Model Core Curriculum PDF was uploaded as reference material for the custom GPT.
5. The custom GPT was instructed to classify textual units deductively according to the Model Core Curriculum hierarchy.
6. Textual units were processed in batches of approximately 20 units.
7. The GPT generated candidate first-tier domains, lower-tier codes, code existence checks, and supplementary notes.
8. Human researchers reviewed the GPT output against the original deidentified textual units and the MCC coding framework.
9. When obvious misclassification was identified, researchers provided specific corrective prompts, such as instructions to recheck PS-03 classifications.
10. The revised output was reviewed again by human researchers.
11. Final coding decisions were made by human researchers.

AI-generated candidate codes were used only as preliminary support. After the AI-assisted preliminary sorting and candidate-coding step, two researchers reviewed the candidate codes, checked the original deidentified textual units, and determined the final MCC codes. Disagreements or uncertainties were resolved through discussion. Thus, the final analytic dataset was based on human-verified coding.

A total of 4097 textual units were extracted from the reflection sheets. For the AI–human concordance analysis, duplicate coding records derived from the same textual unit were collapsed into a single analyzable unit when the same sentence or meaning unit had been represented by multiple code records. Units for AI-generated candidate codes and human coding decisions that could be directly compared were included in this analysis. As a result, 3752 unique analyzable units were used for the AI–human concordance analysis.

Initial AI–human concordance was assessed at three levels of MCC code granularity. At the third-tier MCC code level, complete agreement was 24.2% (909/3752), and at least one code overlapped in 37.4% (1404/3752). At the second-tier MCC code level, complete agreement was 44.6% (1675/3752), and at least one code overlapped at 61.8% (2319/3752). At the first-tier domain level, complete agreement was 58.7% (2202/3752), and at least one code overlapped at 76.8% (2880/3752). These initial concordance metrics characterized the performance of AI-generated candidate codes. Final coding reliability was assessed between the two reviewers. Inter-rater agreement for the final MCC-based coding was excellent (κ=0.97).

**10. Limitations of the AI-Assisted Preliminary Workflow**

The AI-assisted preliminary sorting and candidate-coding workflow had several limitations.

The custom GPT could suggest plausible codes, yet could not determine the educational meaning of a textual unit without human judgment. Some textual units were short or abstract, and final classification required interpretation by researchers familiar with the MCC and the educational context. Some lower-tier classifications were initially inaccurate. Particularly, the research team observed that some PS-related textual units were incorrectly classified under PS-03. This was addressed through specific corrective prompts and researchers’ review.

The AI tool could overemphasize keywords. For example, words such as “treatment,” “drug,” “home visit,” “could not,” or “difficult” could lead to inappropriate classification if the broader context was not considered. The custom GPT was instructed to classify based on context and not to overclassify negative expressions as end-of-life care unless the content explicitly referred to it. AI–human concordance was modest, particularly at the detailed third-tier MCC code level. This indicates that generative AI may be useful for preliminary sorting, orientation, and consistency checking; however, granular deductive coding of reflective text should not be delegated to generative AI without human verification.

The AI-assisted preliminary workflow was conducted within a deductive framework based on the MCC. Therefore, the process was not designed to generate new inductive themes outside the curriculum structure. The AI tool did not determine whether a student had actually performed a clinical procedure or examination. Student-reported clinical activities were measured using daily digital logs. Reflection-sheet coding described written learning content and was not used as direct validation of the digital log data. AI output was used only as preliminary support. All final coding decisions and interpretations were made by the researchers. Time savings, workload reduction, usability, and coding efficiency were not measured prospectively.

## References

1. Medical Education Model Core Curriculum Coordination Committee; Medical Education Model Core Curriculum Expert Research Committee. Model Core Curriculum for Medical Education: AY2022 Revision. Tokyo: Ministry of Education, Culture, Sports, Science and Technology; 2023.
2. Bijker R, Merkouris SS, Dowling NA, Rodda SN. ChatGPT for automated qualitative research: content analysis. J Med Internet Res. 2024;26:e59050.
3. Yue Y, Liu D, Lv Y, Hao J, Cui P. A practical guide and assessment on using ChatGPT to conduct grounded theory: tutorial. J Med Internet Res. 2025;27:e70122.
