# Supplementary material for: Using Student-Reported Digital Daily Logs and Human-Verified AI-Assisted Reflection Coding to Describe Clinical Experiences in Community-Based Clerkships: Exploratory Observational Mixed Methods Study
Source: JMIR Form Res. 2026 Sep 29;10:e102390. doi: 10.2196/102390 (PMC13662440; doi:10.2196/102390)
Supplement: Supplementary file 5 [file formative_v10i1e102390_app6.docx]

**Exploratory Sensitivity Analyses for Contextual Confounding**

**A. Overlap between the presence of university-FD-program-trained CAPs and medically underserved placement**

| **University FD-program site category** | **Not medically underserved, n** | **Medically underserved, n** | **Total, n** |
| --- | --- | --- | --- |
| Without university-FD CAPs | 74 | 4 | 78 |
| With university-FD CAPs | 1 | 33 | 34 |
| Total | 75 | 37 | 112 |

**B. Descriptive comparison of the primary outcome by university FD-program site category**

Primary outcome: number of student-reported examination-based, lower-risk clinical activity types recorded as performed at least once during the 3-week clerkship.

| **Site category** | **n** | **Median** | **IQR** |
| --- | --- | --- | --- |
| Without university-FD CAPs | 78 | 5 | 2-8 |
| With university-FD CAPs | 34 | 10 | 5-12 |

**C. Poisson regression models with robust standard errors**

| **Model** | **Main variable** | **IRR** | **95% CI** | ***P*** |
| --- | --- | --- | --- | --- |
| Model 1: University FD-program model | Presence of university-FD-program-trained CAPs | 1.24 | 0.97-1.58 | .09 |
| Model 1: University FD-program model | Home-visit exposure | 1.47 | 1.08-2.01 | .01 |
| Model 1: University FD-program model | Baseline rural self-efficacy, per SD | 1.17 | 1.03-1.32 | .01 |
| Model 2: Contextual model | Medically underserved placement | 1.31 | 1.02-1.68 | .04 |
| Model 2: Contextual model | Home-visit exposure | 1.45 | 1.06-2.00 | .02 |
| Model 2: Contextual model | Baseline rural self-efficacy, per SD | 1.16 | 1.03-1.31 | .02 |
| Model 3: University FD-program plus medically underserved placement | Presence of university-FD-program-trained CAPs | 0.93 | 0.53-1.64 | .80 |
| Model 3: University FD-program plus medically underserved placement | Medically underserved placement | 1.39 | 0.78-2.48 | .26 |

**D. Negative binomial sensitivity models**

| **Model** | **Main variable** | **IRR** | **95% CI** | ***P*** |
| --- | --- | --- | --- | --- |
| Model 1: University FD-program model | Presence of university-FD-program-trained CAPs | 1.26 | 0.95-1.68 | .11 |
| Model 1: University FD-program model | Home-visit exposure | 1.49 | 1.12-1.97 | .006 |
| Model 1: University FD-program model | Baseline rural self-efficacy, per SD | 1.21 | 1.05-1.38 | .008 |
| Model 2: Contextual model | Medically underserved placement | 1.33 | 1.01-1.74 | .04 |
| Model 2: Contextual model | Home-visit exposure | 1.48 | 1.12-1.95 | .006 |
| Model 2: Contextual model | Baseline rural self-efficacy, per SD | 1.20 | 1.04-1.37 | .01 |
| Model 3: University FD-program plus medically underserved placement | Presence of university-FD-program-trained CAPs | 0.96 | 0.57-1.61 | .87 |
| Model 3: University FD-program plus medically underserved placement | Medically underserved placement | 1.37 | 0.83-2.27 | .22 |

**E. Descriptive comparison among students placed in medically underserved areas**

| **Site category among medically underserved placements** | **n** | **Examination-based, lower-risk clinical activities, median (IQR)** | **Basic clinical and emergency procedures, median (IQR)** | **Total clinical activity types, median (IQR)** |
| --- | --- | --- | --- | --- |
| Without university-FD CAPs | 4 | 9.5 | 8.5 | 17.5 |
| With university-FD CAPs | 33 | 10.0 | 5.0 | 13.0 |

**F. Classification-independent sensitivity analysis across all 50 clinical activity items**

Outcome: total number of student-reported clinical activity types recorded as performed at least once across all 50 MCC-based items.

| **Site category** | **n** | **Median** | **IQR** |
| --- | --- | --- | --- |
| Without university-FD CAPs | 78 | 9 | 5-13 |
| With university-FD CAPs | 34 | 13.5 | 9-18 |

Mann-Whitney U=1850.5; *P* < .001; rank-biserial correlation r = 0.40 (95% CI 0.14-0.60). This analysis did not apply the author-defined 23/27-item classification.

**Notes.** *Sites with university-FD-program-trained CAPs included 34 students; sites without university-FD-program-trained CAPs included 78 students. The latter category does not indicate absence of educational training. The primary outcome was the number of student-reported examination-based, lower-risk clinical activity types recorded as performed at least once during the 3-week clerkship. IRR indicates incidence rate ratio. CI indicates confidence interval. SD indicates standard deviation. CAP is an abbreviation for community-based attending physician.*

Models 1 and 2 were adjusted for home-visit exposure, baseline rural self-efficacy, regional quota admission, sex, and site type. Model 1 included the presence of university-FD-program-trained CAPs as the main exposure. Model 2 replaced this variable with medically underserved placement. Model 3 included both variables to assess estimate instability caused by sparse discordant cases. These models were exploratory sensitivity analyses. Since only 1 student at a site with a university-FD-program-trained CAP was placed outside a medically underserved area and only 4 students at sites without university-FD-program-trained CAPs were placed in medically underserved areas, estimates separating the two variables should be interpreted with caution.
